# Ciprofloxacin-Loaded Gold Nanoparticles against Antimicrobial Resistance: An In Vivo Assessment

**DOI:** 10.3390/nano11113152

**Published:** 2021-11-22

**Authors:** Afrah Nawaz, Syed Mohsin Ali, Nosheen Fatima Rana, Tahreem Tanweer, Amna Batool, Thomas J. Webster, Farid Menaa, Sundus Riaz, Zahra Rehman, Farhat Batool, Misha Fatima, Tuba Maryam, Iqra Shafique, Abida Saleem, Arfa Iqbal

**Affiliations:** 1 Department of Biomedical Engineering and Sciences, School of Mechanical & Manufacturing Engineering, National University of Science & Technology, Islamabad 44000, Pakistan; anawaz.bmes16smme@student.nust.edu.pk (A.N.); sali.bmes17smme@student.nust.edu.pk (S.M.A.); ttanveer.pg@smme.edu.pk (T.T.); amnabatoolx@gmail.com (A.B.); sundusriaz@parc.gov.pk (S.R.); zrehman.bms20smme@student.nust.edu.pk (Z.R.); farhat.bms20smme@student.nust.edu.pk (F.B.); mfatima.bms20smme@student.nust.edu.pk (M.F.); tuba.bms20smme@student.nust.edu.pk (T.M.); ishafique.phd21smme@student.nust.edu.pk (I.S.); asaleem.phd21smme@student.nust.edu.pk (A.S.); aiqbal.bmes19smme@student.nust.edu.pk (A.I.); 2 Department of Chemical Engineering, Northeastern University, Boston, MA 02115, USA; websterthomas02@gmail.com; 3 Departments of Internal Medicine and Nanomedicine, California Innovations Corporation, San Diego, CA 92037, USA; dr.fmenaa@gmail.com

**Keywords:** drug delivery, antibiotics, antimicrobial resistance, gold nanoparticles, ciprofloxacin, *Enterococcus faecalis*, liver and kidney infections, nanotechnology

## Abstract

Metallic nanoparticles, such as gold nanoparticles (AuNPs), have been extensively studied as drug delivery systems for various therapeutic applications. However, drug-loaded-AuNPs have been rarely explored in vivo for their effect on bacteria residing inside tissues. Ciprofloxacin (CIP) is a second-generation fluoroquinolone with a broad-spectrum of antibiotic properties devoid of developing bacteria resistance. This research is focused on the synthesis and physical characterization of Ciprofloxacin-loaded gold nanoparticles (CIP-AuNPs) and their effect on the colonization of *Enterococcus faecalis* in the liver and kidneys of mice. The successfully prepared CIP-AuNPs were stable and exerted enhanced in vitro antibacterial activity against *E. faecalis* compared with free CIP. The optimized CIP-AuNPs were administered (500 µg/Kg) once a day via tail vein to infected mice for eight days and were found to be effective in eradicating *E. faecalis* from the host tissues. Moreover, unlike CIP, CIP-AuNPs were non-hemolytic. In summary, this study demonstrated that CIP-AuNPs are promising and biocompatible alternative therapeutics for *E.-faecalis*-induced infections resistant to conventional drugs (e.g., beta-lactams and vancomycin) and should be further investigated.

## 1. Introduction

*E. faecalis* has become one of the most daunting bacteria among the opportunistic pathogens [1]. This bacterium is responsible for a major percentage of nosocomial infections. It can cause infections in the gastrointestinal, respiratory, and urinary tracts; root canal; etc. Its characteristic features include extraordinary survival capacities in extreme conditions and development of resistance against antibiotics [2,3,4] such as β–lactam antibiotics and vancomycin [5]. Hence, innovative and effective antibiotics are required to tackle drug resistance mediated by *E. faecalis* infections.

The development of antimicrobial resistance (AMR) is one of the most pressing global issues, and great efforts have been committed to explore new and advanced antibiotics. However, extensive and impeded regulatory approval has shifted the focus to improve the efficacy of existing blockbuster drugs [6]. Advances in nanotechnology offer exciting methods to provide novel materials that can support penetration into biofilms, promote bacteria entry of the drugs, and present synergism with their own functional attributes [7,8,9]. Metallic nanoparticles (NPs), such as gold nanoparticles (AuNPs), are gaining recognition as improved drug delivery vehicles. They have shown effectiveness for various medical illnesses, including cancer, chronic diseases, and antibacterial, antiviral, and antifungal infections [10]. AuNPs do not exert (inherent) antibacterial activity; however, they enhance antibacterial properties of the loaded antibacterial drugs [11]. Drug-loaded AuNPs collapse bacteria membrane potential, hinder ATPase activity, and halt binding of ribosomes to tRNA leading to deteriorated bacterial cell metabolism [12]. Moreover, their site directed activity can target biofilms and quorum sensing [13,14]. The stability and the activity of such AuNPs under physiological conditions (such as pH, temperature, etc.) have also been evaluated in various studies, as such factors can modify their antibacterial activity [15,16]. Over the last years, various nanocarrier systems against *E. faecalis* have been reported such as ciprofloxacin and metronidazole encapsulated nanomatrix gels [17], bismuth nanoparticles [18], chitosan–propolis nanoparticles [19], doxycycline-functionalized polymeric nanoparticles [20], methylene blue and biogenic gold nanoparticles [21], silver nanoparticles and calcium hydroxide mixtures [22], biosynthesized silver nanoparticles [23], and graphene oxide nanoparticles [24].

Ciprofloxacin [1-cyclopropyl-6-fluoro-1,4-dihydro-4-oxo-7 piperazinylquinolone-3-carboxylic acid] (CIP) is a large spectrum fluoroquinolone antibiotic that is widely used for the treatment of numerous bacterial infections in joints, bones, skin, tooth, gastrointestinal, and urinary and respiratory tracts [25]. CIP-loaded gold nanoparticles (CIP-AuNPs) were reported by Tom et al. in 2004 [26]. Recently reported nanocarriers for CIP include CIP-loaded nanocomposites [27], PLGA–chitosan-based ciprofloxacin [28], Ciprofloxacin-containing polymer-based nanofibers (scaffolds) [29], and CIP-loaded single-walled carbon nanotubes [30]. This study aims to use ciprofloxacin protected gold nanoparticles to eradicate bacteria in the tissues of mice. The physical characterization of the nanoformulated version of this drug along with in vitro antibacterial activity, against *E. faecalis,* were carried out. For in vivo studies, an infectious mouse model was developed for *E. faecalis* and anti-colonization through the use of CIP-AuNPs was determined.

## 2. Materials and Methods

### 2.1. Bacterial Strains

The Gram-positive bacterial strain used was *E. faecalis* JH2-2 (derived from the parental strain JH2). GM17 medium was used for their growth at 37 °C [31].

### 2.2. Preparation of AuNPs and CIP-AuNPs

The AuNPs were prepared by a previously reported method [26]. Trisodium citrate (0.5 mM) and chloroauric acid (0.5 mM) (MERK, Munich, Germany) were fluxed together. Citrate acted as a stabilizer as well as a reducing agent. The particle formation was confirmed upon achieving a red wine color. For the loading of CIP on AuNPs, 20 mL of the above solution was mixed with 5 mL of CIP (0.5, 1.0, 1.5, 2, and 2.5 mM) at pH 6.5. Then, the solution was stirred until the red color turned to blue–purple.

### 2.3. Characterization of AuNPs and CIP-AuNPs

The CIP-AuNPs were characterized by UV–Vis spectrophotometry using a UV-2800 (BMS Biotechnology Medical Services, Madrid, Spain) spectrophotometer. Zeta potential was recorded using a Malvern Zeta sizer (Malvern, UK). Scanning electron microscopy (SEM) and Energy-dispersive X-ray spectroscopy (EDS) analyses were carried out using a SEM VEG 3 LMU (Tescan, Czech Republic), while Fourier-Transform Infrared Spectroscopy (FTIR) analyses were carried out using a Bruker FTIR Spectrometer ALPHA II (Westborough, MA, USA).

### 2.4. Drug Loading Capacity and Encapsulation Efficiency

CIP loading capacity and encapsulation efficiency by the AuNPs were evaluated using Equations (1) and (2), respectively.

(1) $$\mathrm{Loading}\;\mathrm{capacity}\;\left(\%\right)=\frac{\mathrm{Weight}\;\mathrm{of}\;\mathrm{CIP}\;\mathrm{in}\;\mathrm{CIP}-\mathrm{AuNPs}}{\mathrm{Weight}\;\mathrm{of}\;\mathrm{CIP}-\mathrm{AuNPs}}\times 100\;$$

(2) $$\mathrm{Encapsulation}\;\mathrm{Efficiency}\;\left(\%\right)=\frac{\mathrm{Total}\;\mathrm{CIP}\;\mathrm{added}\;-\;\mathrm{Free}\;\mathrm{CIP}\;}{\mathrm{Total}\;\mathrm{CIP}\;\mathrm{added}}\times 100$$

### 2.5. Drug Release Efficiency

The in vitro release of CIP from the CIP-AuNPs formulated with various concentrations of CIP was studied over time using a UV–Vis spectrophotometer at 280 nm from 0 to 24 h (every 2 h) by adding 20 mL of a 20-mM PBS buffer to 20 mL of the CIP-AuNPs solutions. The cumulative drug release was calculated using Equation (3).

(3) $$\mathrm{Cumulative}\;\mathrm{drug}\;\mathrm{release}\;\left(\%\right)=\frac{\mathrm{CIP}\;\mathrm{released}\;\mathrm{from}\;\mathrm{the}\;\mathrm{CIP}-\mathrm{AuNPs}\;\mathrm{at}\;t\;}{\;\mathrm{The}\;\mathrm{total}\;\mathrm{amount}\;\mathrm{of}\;\mathrm{CIP}\;\mathrm{loaded}\;\mathrm{onto}\;\mathrm{the}\;\mathrm{AuNPs}.\;}$$

To determine the quantity of the drug present at the absorption site, a significant predictor T_60%_, was calculated as the time taken to release 60% of the drug. For instance, Stineman interpolation using Minitab 17 software was applied. The T_60%_ drug release data were fit into the Korsmeyer-Peppas model for non-swellable matrices (Section S1.3).

### 2.6. Kinetic Analysis of the Drug Release

To determine the drug release from the CIP-AuNPs, numerous mathematical models (zero-order, first-order, and Higuchi’s model) were employed (Section S1.3) [27,32].

### 2.7. In Vitro Stability of CIP-AuNPs

The effect of temperature on CIP-AuNPs was found by heating CIP-AuNPs at different temperatures (25 °C, 50 °C, 75 °C, and 100 °C) for 30 min. The effect of pH values (4, 7, and 10) and different salt concentrations (0.05, 0.1, 0.5, and 1 M) on CIP-AuNPs were also determined. For salt concentration, 10 mL of CIP-AuNPs were centrifuged at 10,000× *g* for 10 min. The resulting pellets were suspended in NaCl solutions (50 mM–1 M) at 37 °C for 24 h.

### 2.8. In Vitro Antibacterial Potential of CIP-AuNPs

The Minimum Inhibitory Concentration (MIC) of free CIP was calculated using different concentrations of CIP and CIP-AuNPs (0.1–10 mg/mL) against *E. faecalis.* The bacterial cultures at exponential phase of OD_600_ were harvested and counted for 10^6^ CFU/mL using the standard dilution method. Using a standard inoculum of 10^6^ CFUs, cultures were incubated at standard conditions for 24 h. The concentrations with a 50% reduction in bacterial count were observed as MIC. With an inoculum of approximately 10^6^ CFU/mL, 50 µL of a 10-fold-diluted culture were plated on M-17 agar plates for measuring the viable cells. Colonies were counted after 24 h [33,34]. Moreover, a zone of inhibition test was also conducted by using the disc diffusion method to compare the antibacterial activity of CIP-AuNPs and free CIP. An *E. faecalis* culture was developed on the nutrient agar plate. A zone of inhibition (in mm) was then measured for *E. faecalis* using the Kirby–Bauer method. Sterile Whatman filter paper discs were impregnated with CIP, AuNPs, and CIP-AuNPs at a concentration of 10 µg/disc each.

### 2.9. Hemolytic Activity of CIP-AuNPs

A hemolysis test was carried out to compare the hemolytic activity of different concentrations (i.e., 10, 25, 50, and 100 µg/mL) of CIP-AuNPs (2 mM CIP), AuNPs, and CIP. Blood samples were taken from healthy female donors. Red blood cells were incubated for 4 h using the method described by Zarmina et al. [35]. As a positive control, Triton X-100 (0.5%) was used, while PBS was used as a negative control. The absorbance was measured at a wavelength of 550 nm.

### 2.10. Colonization of E. faecalis in BALB/c Mice

The in vivo investigation was conducted using female *BALB/c* mice (eight weeks old, weighing 25–30 g; *n* = 15) purchased from the National Institute of Health (NIH), Islamabad Pakistan. They were kept at 25 ± 2 °C and presented with a natural light–dark cycle (10–14 h). The mice were given autoclaved tap water and a normal diet ad libitum. For bacterial colonization in hepatic and renal tissues, a well-established intravenous (IV) infection model was used [36]. The GM17 broth at a temperature of 37 °C was used and the preculture was grown overnight. A total of 100 µL of preculture was added to brain heart infused (BHI) medium supplemented with 40% filter-sterilized serum. Phosphate buffer saline (PBS), pH 7.4, was then used to wash the subsequent pellets from the cultures, optimized by colony counting for the number of cells. The bacterial pallets were then suspended in sterile PBS. A total of 100 µL of the suspension adjusted for 1 × 10^9^ cells/mL of bacterial suspensions were (tail vein) injected into each of the female mice (*n* = 15).

### 2.11. In Vivo Antibacterial Activity of CIP-AuNPs

To assess the in vivo antibacterial activity of CIP-AuNPs and CIP, they were suspended or dissolved in PBS buffer. The infected group (*n* = 15) was treated with CIP-AuNPs (500 µg/Kg, *n* = 5) and with free CIP (10 mg/Kg, *n* = 5); the remaining five mice remained untreated. CIP-AuNPs and free CIP were delivered by the tail vein once a day for eight days starting from the seventh day of infection until the day of the challenge. After a week of treatment, all mice were sacrificed, and their liver and kidneys were removed to measure the viable bacterial count through the colony-forming unit (CFU) method. For instance, organs were weighed and homogenized in 10 mL of a PBS solution. 10-fold dilutions of the homogenate were plated on the agar plate. CFUs were counted after 24 h.

### 2.12. Statistical Analysis

Statistical analysis (such as the mean, standard deviation, and multiple group comparison analysis by one-way ANOVA) was carried out using Origin 2021 (OriginLab Corporation, Northampton, MA, USA) and Graph Pad Prism 9.2.

## 3. Results

### 3.1. Synthesis of AuNPS and CIP-AuNPs

**Figure 1:**
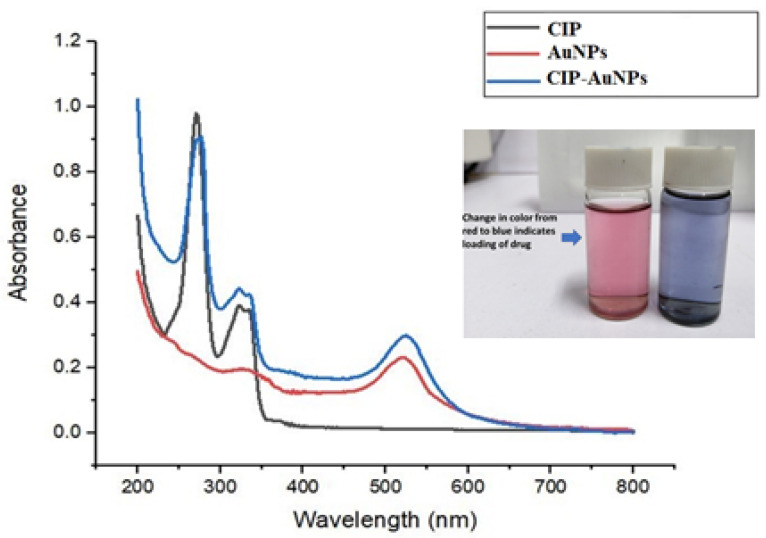
UV–Vis spectra of CIP, AuNPs, and CIP-AuNPs.

The synthesis of AuNPs was accomplished by a chemical reduction method. The red color confirmed the formation of AuNPs upon the addition of citrate to chloroauric acid. The UV–Vis graph shows the absorbance peak of gold nanoparticles at 521 nm (Figure 1).

**Figure 2:**
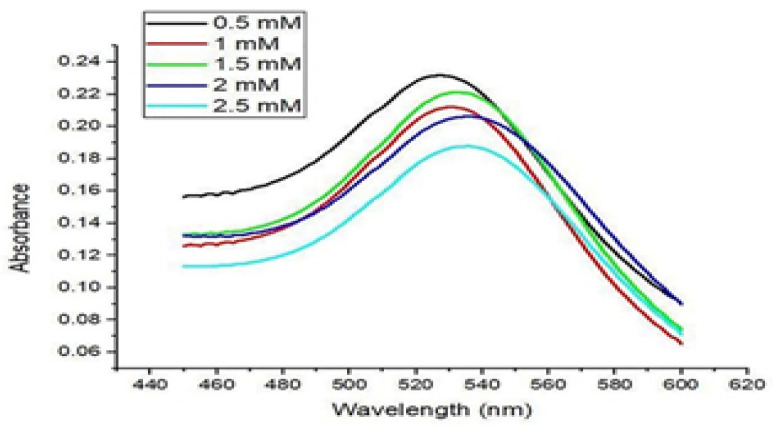
UV–Vis spectra of CIP-AuNPs loaded with various CIP concentrations.

Further, the addition of CIP into the AuNP solution resulted in a purple to bluish solution, indicating CIP adsorption onto the AuNPs. UV–Vis spectroscopy verified the adsorption of various CIP concentrations on AuNPs, as presented in Figure 2. The transition of the solution color took place upon adding different CIP concentrations, typically from red to purple to bluish, and finally, to dark blue (Figure 1).

### 3.2. CIP Encapsulation Efficiency and CIP Loading Capacity

CIP encapsulation efficiency was the highest (60.83%) at the highest CIP concentration used (2.5 mM); conversely, CIP encapsulation efficiency into AuNPs was the lowest (24.43%) at the lowest CIP concentration used (0.5 mM) (Table 1). Consistently, the drug loading capacity was also drug-concentration-dependent. Indeed, CIP loading capacity into AuNPs was the highest (34.54%) at the highest CIP concentration used (2.5 mM); conversely, CIP loading capacity into AuNPs was the lowest (8.85%) at the lowest CIP concentration (0.5 mM) (Table 1).

### 3.3. Particle Size and Zeta Potential of AuNPs and CIP-AuNPs

The average particle size (PS) of AuNPs was found to be 23 nm. The PS for the different CIP-AuNPs are mentioned in Table 2. Upon addition of CIP (0.5 mM and 1 mM), the size of the NPs remained almost the same at 24 nm. The PS increased to 41, 88, and 128 nm using 1.5, 2.0, and 2.5 mM CIP, respectively.

The zeta size and charge values of the CIP-AuNPs and AuNPs are mentioned in Table 2. The AuNP had a negative charge of −32.1 mV, which remained unchanged upon the addition of 0.5 mM CIP. However, the zeta potential (ZP) and PDI values of the CIP-AuNPs at a 1.5 mM CIP concentration were −19.7 ± 6.65 mV and 0.680, respectively.

### 3.4. Surface Morphology and Elemental Chemical Composition of AuNPs by SEM–EDS

**Figure 3:**
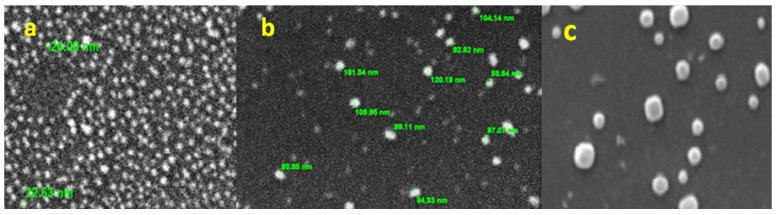
SEM micrographs of (**a**) AuNPs, (**b**) AuNPs (2 mM), and (**c**) CIP-AuNPs (2.5 mM).

The surface morphology of the chemically prepared NPs (AuNPs and CIP-AuNPs) was analyzed by SEM at 10 kV. SEM images revealed spherically-shaped AuNPs (Figure 3a), 2 mM CIP-AuNPs (Figure 3b), and 2.5 mM CIP-AuNPs (Figure 3c). The SEM analysis further presented a high polydispersity of 2.5 mM CIP-AuNPs.

Besides, EDX analysis was carried out for elemental mapping of CIP-AuNPs (2.5 mM) (Supplementary Materials Figure S1, Section S2.1).

### 3.5. Structural Analysis of CIP-AuNPs by FTIR Spectroscopy

**Figure 4:**
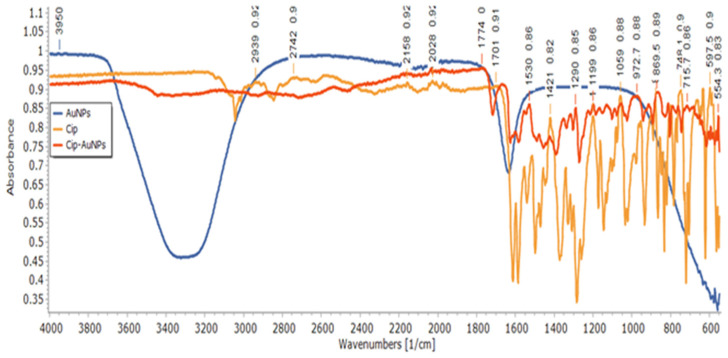
FTIR spectra of CIP-AuNPs, AuNPs, and free CIP.

The FTIR of CIP-AuNPs, AuNPs, and free CIP are presented in Figure 4 and Supplementary Materials Table S1. FTIR results showed the successful adsorption of CIP in AuNPs. The stretching of the N–H bond of the imino moiety on the piperazine group of the CIP was shown by the band at 3410 cm^−1^. Absorption bands at 1655 cm^−1^ and 1070 cm^−1^ represent a primary amine (N–H) bend of the pyridone moiety and the C–F functional group of the CIP, respectively [30]. The peak at 1634.84 cm^−1^ corresponded to C = O symmetric stretching, and the band at 1381.02 cm^−1^ was assigned to the C–H stretching vibration in the AuNPs. The CIP-AuNPs exhibited bands at 3341 cm^−1^, which corresponded to the strong stretching vibration of the O–H group of alcohols and phenols, while the band at 2062 cm^−1^ referred to strong C–H stretching [33]. The stretching bands at 3410 cm^−1^ of the N–H bond of the imino moiety on the piperazine group of the CIP moved to 3449 cm^−1^ for the CIP-AuNPs, showing adsorption of CIP on AuNPs. It has been reported that the nitrogen atom of the NH moiety of the piperazine group of CIP binds to the AuNP surface to form CIP-AuNPs. The binding of CIP to the surface of AuNPs was confirmed by the fact that the corresponding NH_2_ peaks were broadened and shifted to higher wavelengths (1655 cm^−1^ to 1667 cm^−1^). The broad stretching band of O–H was due to water traces in the CIP-AuNP and AuNP samples. The weak bands around 2900 cm^−1^ in CIP-AuNPs are explained by traces of citrate. 

### 3.6. Kinetics of CIP Drug Release from AuNPs

**Figure 5:**
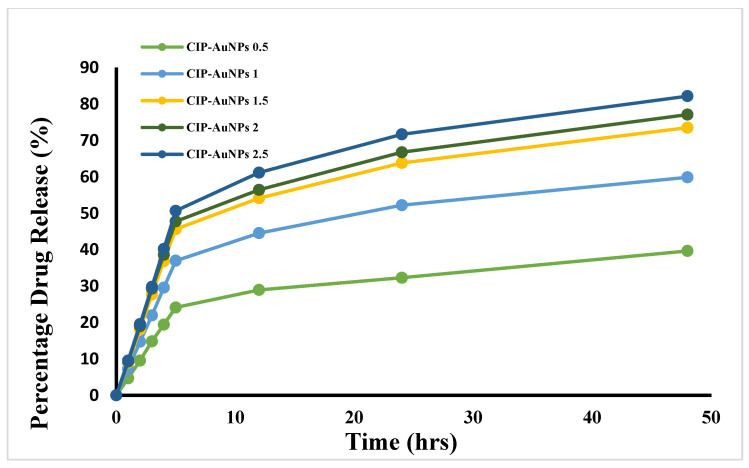
In vitro CIP release from AuNPs loaded with various CIP concentrations.

The drug release curve is represented by a normal biphasic drug release pattern, which can be split into two stages (Figure 5). In the initial stage (0–5 h), a rapid release of CIP occurred from all the formulations. This confirmed the adsorption of CIP on the surface of AuNPs, in which the CIP-AuNPs displayed a high surface-to-volume ratio due to their small size, which promoted burst release [34]. The second stage (5–24 h) was when the drug was released in a prolonged fashion. The T_60_ of CIP release from the AuNPs loaded with 2 mM of CIP was obtained at 27 h. The T_60_ for 0.5 mM CIP, 1.5 mM CIP, or 2.5 mM CIP was found to be around 70 at 38 h, 29 h, and 24.5 h, respectively, upon extrapolating the drug release. These results indicated that drug release was seed size-dependent i.e., the smaller the nanoparticles, the slower the drug release.

A first-order release was observed for CIP release from the CIP-AuNPs, which demonstrated that CIP release from CIP-AuNPs (2 mM) was concentration-dependent (Supplementary Materials Figures S2–S6). Overall, the release studies demonstrated that CIP-AuNPs loaded with >1 mM CIP are suitable nanoformulations for CIP administration in a sustained manner. The initial burst release will act as a loading dose to suppress the disease spread, while the sustained release phase will contribute towards a better therapeutic effect.

### 3.7. Stability Tests on CIP-AuNPs

The CIP-AuNPs with targeted drug concentration (2 mM) showed stability at room temperature and in a basic medium. They were unstable at different salt concentrations. The color change from blue to dark blue was observed with an increase in temperature for the CIP-AuNPs, and 2.0 and 2.5 mM of CIP (Supplementary Materials Figures S7–S11), which is attributed to the increase in particle size (PS) due to aggregation. CIP-AuNPs (2 mM) were stable at room temperature (25 °C), and the A_max_ increases on the order of 25 °C < 50 °C < 75 °C < 100 °C. With different pH values (Supplementary Materials Figures S12–S16) and increasing salt concentrations (Supplementary Materials Figures S17–S21), irregular trends in A_max_ (adsorption peak of CIP, 280 nm) of CIP-AuNPs in all formulations were observed. For further analysis, the CIP-AuNPs (2 mM) formulation was selected.

### 3.8. In Vitro Antibacterial Activity of CIP-AuNPs at the Optimized Dose

**Figure 6:**
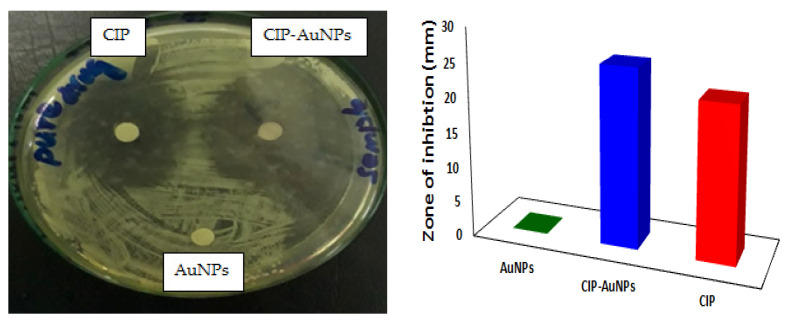
The zones of inhibition (ZI) displayed by CIP, AuNPs, and CIP-AuNPs for *E. faecalis*.

The MICs of free CIP and CIP-AuNPs were tested against *E. faecalis* JH2-2. For comparison of antibacterial activity of CIP and CIP-AuNPs (2 mM), the Minimum Inhibitory Concentration was obtained for our strain. The MIC of CIP and CIP-AuNPs were found to be 2 µg/mL and 1 µg/mL, respectively. The zone of inhibition using 10 µg/disc are presented in Figure 6. As such, a larger zone of inhibition of 23 mm was obtained for the CIP-AuNPs compared with CIP (21 mm), whereas no zone of inhibition was obtained for the bare AuNPs.

### 3.9. In Vivo Anticolonizing Potential of CIP-AuNPs in an Animal Model

**Figure 7:**
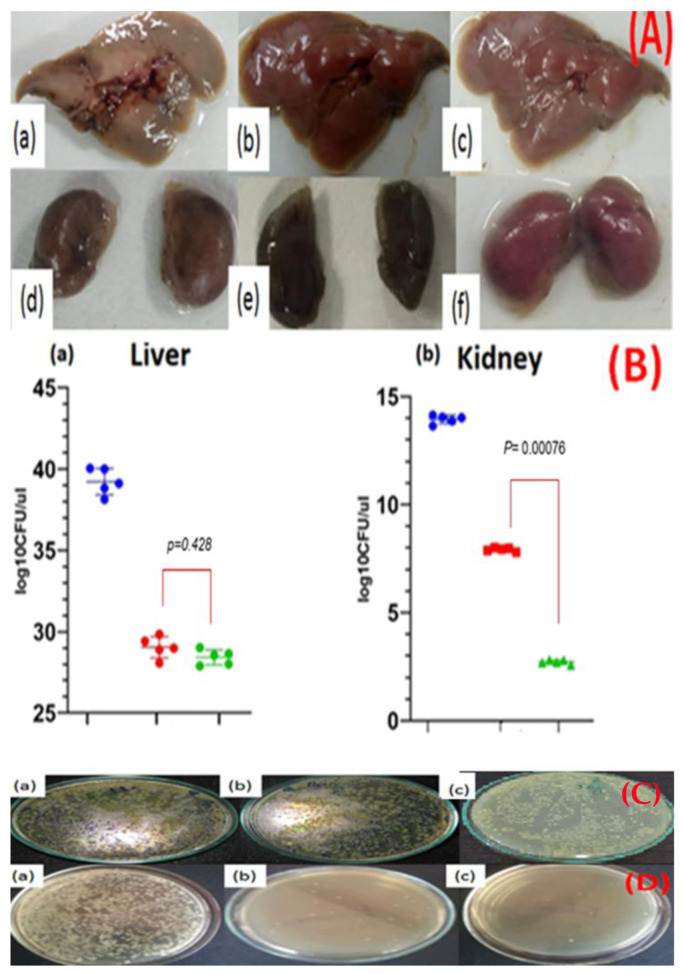
Effect of CIP (10 mg/Kg) and CIP-AuNPs (500 µg/Kg) on the colonization of *E. faecalis* in the (**A**) mouse organs—(**a**) infected liver, (**b**) CIP-treated liver, (**c**) CIP-AuNPs-treated liver, (**d**) infected kidney, (**e**) CIP-treated kidney, (**f**) CIP-AuNPs treated kidneys, infected kidneys, (**B**(**a**,**b**)) CFU *E. faecalis* in the infected liver and kidney; (**C**) CFU counting in the liver—(**a**) infected, (**b**) CIP-treated, and (**c**) CIP-AuNPs-treated; (**D**) CFU counting in the kidneys—(**a**) infected, (**b**) CIP-treated, and (**c**) CIP-AuNPs-treated.

The infected mouse liver and kidneys appeared to be inflamed and some lighter zones underneath the liver (Figure 7A) were observed. Since the strain was not biofilm-forming, no particular lesions were visualized in the infected liver Figure 7A(a). Mice treated with CIP and CIP-AuNPs had a normal liver appearance Figure 7A(b,c) respectively. The kidneys of infected, CIP treated and CIP-AuNPs treated mice presented no particular change in morphology or texture, Figure 7A(d–f) respectively. The average bacterial count (log_10_CFU/gm organ) in the infected liver was 39.53± 1.93 (Figure 7B(a)). CIP and CIP-AuNPs treated mice harbored 29.049 ± 1.343 log_10_CFU/mL and 28.4 ± 0.421 log_10_CFU/mL (*p* = 0.428) respectively, in the liver. The bacterial load in the kidneys of infected mice was 13.98 ± 0.232 log_10_CFU/mL (Figure 7B(b)) while kidneys of CIP and CIP-AuNPs treated mice presented significantly lower bacterial load, 7.91 ± 0.2132.76 ± 0.034 log_10_CFU/mL (*p* < 0.0D0076), respectively. The in vivo data revealed that the mice treated with CIP-AuNPs significantly reduced bacterial colonization in the kidneys (Figure 7B,D) compared with those of CIP-treated mice. The CFU for the 10-fold serial dilutions in the infected, CIP treated and CIP AuNPs treated liver are shown in Figure 7C(a–c) respectively. Same dilutions for infected, CIP treated and CIP AuNPs treated kidneys are shown in Figure 7D(a–c) respectively.

### 3.10. Hemolytic Activity of CIP-AuNPs

**Figure 8:**
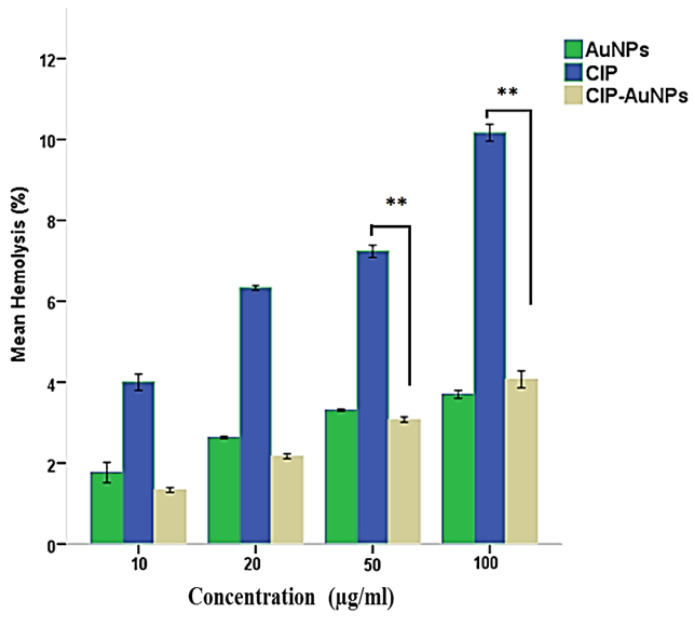
Hemolytic activity of CIP, AuNPs, and CIP-AuNPs, ** presents *p* ≤ 0.01.

Hemolytic activity results of the CIP, AuNPs, and CIP-AuNPs are presented in Figure 8. According to ISO/TR 7406, the percentage hemolysis considered to be safe is <5%. These results present the greater hemolytic activity of CIP (6.4, 7.2 and 10%) compared with AuNPs and CIP-AuNPs (2 mM). Since the intravenous route gave an escape to the drug from first-pass metabolism, results indicate a hundred percent availability of the drug or nanoparticles in plasma. Considering the average mouse weight was 28 g, the average injected doses of CIP and CIP-AuNPs in the mice were 280 µg and 14 µg, respectively. The results indicated that CIP had hemolytic activity at concentrations of 20 µg/mL and above.

## 4. Discussion

Ciprofloxacin is a second-generation fluoroquinolone that is active against many Gram-negative and Gram-positive bacteria. It acts through inhibition of bacterial DNA gyrase and topoisomerase IV. There has been no cross-resistance reported for CIP and other fluoroquinolones; therefore, it is of high clinical value. However, there are certain side effects associated with CIP including low blood sugar, headache, nerve damage causing numbness, tendon rupture, and joint pains. Gold nanoparticles are efficient drug carriers that have the ability to improve the antibacterial effects of loaded antibiotics as well as to reduce the amount of drug required to be effective because of their retention and penetration into bacterial biofilms and cell membranes at the infected sites.

This study reported the effectiveness of spherical CIP-AuNPs as an antibacterial platform against *E. faecalis* infection in the kidneys and liver of mice. The spherical CIP-AuNPs (Figure 1 and Figure 3) were successfully prepared using a non-simple ionic interaction between CIP and negatively charged AuNPs, which maintained their therapeutic functions without chemical modification [28]. Drug adsorption or loading on the NP was determined by UV–Vis spectroscopy and FTIR. In CIP-AuNPs, the zeta potential values changing from −32.1 mV to −19.7 mV and −13.4 mV indicated the adsorption of CIP onto AuNPs [37]. The negative charge on the AuNPs and the positive charge of the CIP [26] (at 6.5 pH) resulted in electrostatic interactions; hence, enhanced CIP encapsulation efficiency and loading capacity was observed. The addition of different concentrations of the drug to the nanoparticles changed the color from red to purple to bluish purple, and finally, to blue. Increasing the concentration of CIP-AuNPs (>2 mM of CIP concentration) caused the zeta potential values to become less negative (Table 2). The zeta potential values ranging between −5 mV and 5 mV generally demonstrate fast aggregation [38]. The SPR spectra of CIP-AuNPs—2 mM (Figure 1) indicated an increase in the peak intensity of the AuNPs upon the addition of CIP. Moreover, there was a slight shift in the CIP peaks. These changes indicated loading of CIP onto the AuNPs. Considering the standard limits, the zeta potential values of CIP-AuNPs at a 2.5-mM CIP concentration showed fast aggregation. Optimization of the drug for loading is, thus, an important criterion for electrostatically loaded drugs. The encapsulation efficiency, which actually represents the amount of the drug that gets incorporated into a particle at a provided concentration, may help in this regard. The drug loading values present the percentage of drug in terms of weight of the nanoparticles. Considering this, we selected CIP-AuNPs (at a 2 mM CIP concentration) for the antibacterial and hemolysis studies.

The stability testing of the CIP-AuNPs illustrated a drastic shift in the stability of the CIP-AuNPs at 100 °C, which could be due to an increase in collision frequency between CIP-AuNPs. The activation energy produced from these collisions would be enough for CIP-AuNPs to react, which is fully depicted by the Arrhenius equation (Supplementary materials Figures S7–S12). From the observation of the effect of temperature on different CIP-AuNPs, the 2.5 mM ratio showed a change to dark blue. Thus broadening of the CIP-AuNPs peak at the 500 nm range shows particle aggregation. This is due to the fact that the particles destabilize causing a decrease in extinction peak intensity, resulting in a lower number of stable nanoparticles. In the case of pH, the CIP-AuNPs with 2 mM of CIP showed a peak shift from 537 nm to 521 nm at pH 7 and 10 (Figure S15). This is because of the fact that the CIP acquired a negative charge. This negative charge occurred due to deprotonation of the amine group, which then further led to drug unloading. The effect of salt showed the broadening of CIP-AuNPs peaks, this could be attributed to the fact of a very high molar concentrations of the salt used in this experiment. The submolar salt (100 mM and above) (Supplementary materials S17–S21) led to AuNP aggregation and drug unloading.

In the current study, the in vitro drug release was assessed by various kinetic models consisting of the zero-order, first-order, Higuchi, and Korsmeyer–Peppas models. A first-order release was seen for CIP release from the CIP-AuNPs. This shows that CIP release from CIP-AuNPs was directly dependent on the drug concentration (Supplementary materials Figures S2–S6). Overall, the release studies demonstrated the dependence of CIP-AuNPs on particle size. Smaller particles release drugs in a very slow fashion. The most suitable drug release kinetics was obtained by CIP-AuNPs (2 mM). 

A higher level of antimicrobial activity is afforded by the CIP-AuNPs. This is due to the large surface-to-volume ratio of AuNPs, which facilitated the adsorption of CIP molecules onto the AuNP surfaces via electrostatic interaction between the amine groups of CIP and AuNPs. The MIC of the CIP-AuNPs was half to that of CIP. Considering that the overall particle seed was gold, the drug amount administered was much lower; thus, carrier driven therapies require less amount of the drug to achieve the same therapeutic outcome. It is well established that silver nanoparticles prepared via a chemical reduction procedure exhibited antibacterial activity. However, for the AuNPs in this study, no inherent antibacterial activity was obtained. This depicts that the surface-area-to-volume ratio of AuNPs served its purpose as a drug delivery system and supported the CIP approach to the target site, thus improving antibacterial activity. Studies revealed that both positively and negatively charged gold nanoparticles get localized in the bacterial membranes. For entry, the binding of negative charged particles to the bacteria’s lipid bilayer results in local gelation. In contrast, the positively charged NPs get along the fluidity in the gelled regions of the bilayers [39,40,41]. Once CIP-AuNPs have reached the bacterial surface, they might have entered or fused with the cell membrane of *E. faecalis* and served as a reserve for the continuous release of CIP, which must have then diffused into the interior of the *E. faecalis*. It has also been reported that while acquiring resistance, *E. faecalis* has cationic membrane proteins on their surface, which alter the membrane fluidity and potential. At moderate levels of *E. faecalis* resistance, the cell surface becomes neutralized, while at further higher resistance levels, the cell surface acquires a positive charge. This positive charge on the membrane may further permit CIP-AuNPs to interact and enter the bacterial cell [39,40,41]. 

The in vivo antibacterial activity showed significant reduction in CFU by CIP-AuNPs compared with CIP. However, in the case of the liver, the difference was not significant but comparable to CIP; this could be attributed to the fact that the *E. faecalis* presented a very high hepatic load. Moreover, since the nanoparticles did not have a polymer coating such as polyethylene glycol, many of them can be uptaken by the reticuloendothelial system and could have been taken for detoxification. In the case of the kidneys, the nanoparticles may have stayed or been entrapped at the glomerular filtration barrier (GFB), which is present within the glomerulus and sandwiched between the glomerular capillary and Bowman’s space. Different layers of the GFB have pore sizes between 8–100 nm and, thus, act as a sieve for the nanoparticles. The nanoparticles, especially the anionic nanoparticles, have higher retention time in the kidneys and, in general, the nanoparticles disintegrate before passing through the GFB. The accumulation of nanoparticles with this size range could be the reason behind the efficiency of the significant decrease in bacterial count in the mice treated with CIP-AuNPs over mice treated with CIP. 

Ciprofloxacin has also been associated with hemolytic anemia [42]. In addition to improved antibacterial activity, this study (Figure 7) also reported limited hemolytic activity of the CIP-AuNPs (2 mM) on human red blood cells (RBCs). These results presented the greater hemolytic activity of CIP (6.4, 7.2, and 10.6%) as compared to AuNPs and CIP-AuNPs. It has been reported that a positive charge on the particle‘s surface possesses a membrane-destabilizing and concomitantly destructive effect that results from the interaction of a positive charge and a negative charge of the cell membrane. Therefore, the limited hemolytic activity of the CIP-AuNPs (with a 2-mM CIP concentration) is attributable to the negative charge on its surface and the lack of free CIP in the medium. Considering the average mouse weight as 28 g, the average injected doses of CIP and CIP-AuNPs in the mice were 280 µg and 14 µg, respectively. The results indicated CIP to be hemolytic at a concentration of 20 µg/mL and above (Figure 8). Due to this, the mice experienced hyperactivity and irritation at the site of injection soon after the administration of CIP, and decreased eating after the second day of treatment.

Antibiotics, such as CIP, are an attraction because of their broad spectrum and lack of resistance development. *E. faecalis* has resistance acquisition capacity and this challenge can be sealed with CIP. Despite of the enormous work conducted on the development of CIP nanocarriers, there is a lack of studies investigating the effects of these nanoparticles in animal models. These studies are extremely important as bacteria residing in the tissue and forming biofilms might not be easily accessed by circulating nanoparticles and therefore they can obtain a colonization advantage. This in vivo study concluded showing efficiency of the CIP-loaded gold nanoparticles against intra-abdominal and urinary tract infections caused by *E. faecalis*. Further investigations for the biocompatibility and biofilm formation in the host tissues are necessary and will be vital for the further assessment of these novel nanoparticles.

## Figures and Tables

**Table 1:** Encapsulation efficiency (%) and loading capacity (%) of CIP onto the AuNPs at varying CIP concentrations.

CIP Concentration AuNPs	Encapsulation Efficiency (%)	Loading Capacity (%)
0.5 mM	24.43	8.85
1.0 mM	29.30	15.60
1.5 mM	30.65	28.85
2.0 mM	48.92	33.81
2.5 mM	60.83	34.54

**Table 2:** Zeta potential values for CIP-AuNPs, AuNPs, and CIP.

CIP-AuNPs	Z-Average (d. nm)	PDI	St Dev (d. nm)	Zeta Potential (mV)
0.5 mM	24.43	0.26	6.21	−32.1
1.0 mM	24.09	0.301	6.044	−33.3
1.5 mM	41	0.68	10.21	−19.7
2.0 mM	88.2	1.000	57.4	−13.4
2.5 mM	128.2	0.48	79.18	−2.12

## Data Availability

The data presented in this study are available on requests from corresponding author.

## Supplementary Materials

The following are available online at https://www.mdpi.com/article/10.3390/nano11113152/s1. Figure S1: EDX analysis of the CIP-AuNPs; Figure S2: Kinetic analysis of the drug release for CIP-AuNPs (0.5 mM CIP)—(A) zero-order kinetics, (B) Korsmeyer–Peppas plot, (C) first-order kinetics, and (D) Higuchi kinetics; Figure S3: Kinetic analysis of the drug release for CIP-AuNPs (1 mM CIP)—(A) zero-order kinetics, (B) Korsmeyer–Peppas plot, (C) first-order kinetics, and (D) Higuchi kinetics; Figure S4: Kinetic analysis of the drug release for CIP-AuNPs (1.5 mM CIP)—(A) zero-order kinetics, (B) Korsmeyer–Peppas plot, (C) first-order kinetics, and (D) Higuchi kinetics; Figure S5: Kinetic analysis of the drug release for CIP-AuNPs (2 mM CIP)—(A) zero-order kinetics, (B) Korsmeyer–Peppas plot, (C) first-order kinetics, and (D) Higuchi kinetics; Figure S6: Kinetic analysis of the drug release for CIP-AuNPs (2.5 mM CIP)—(A) zero-order kinetics, (B) Korsmeyer–Peppas plot, (C) first-order kinetics, and (D) Higuchi kinetics; Figure S7: Temperature effect on CIP-AuNPs formulated with 0.5 mM CIP; Figure S8: Temperature effect on CIP-AuNPs formulated with 1 mM CIP; Figure S9: Temperature effect on CIP-AuNPs formulated with 1.5 mM CIP; Figure S10. Temperature effect on CIP-AuNPs formulated with a 2-mM CIP concentration; Figure S11: Temperature effect on CIP-AuNPs formulated with 2.5 mM CIP; Figure S12: pH effect on CIP-AuNPs formulated with 0.5 mM CIP; Figure S13: pH effect on CIP-AuNPs formulated with 1 mM CIP; Figure S14: pH effect on CIP-AuNPs formulated with 1.5 mM CIP; Figure S15: pH effect on CIP-AuNPs formulated with 2 mM CIP. Figure S16: pH effect on CIP-AuNPs formulated with 2.5 mM CIP; Figure S17: Effect of salt concentrations on CIP-AuNPs formulated with 0.5 mM CIP; Figure S18: Effect of salt concentrations on CIP-AuNPs formulated with 1 mM CIP; Figure S19: Effect of salt concentrations on CIP-AuNPs formulated with 1.5 mM CIP; Figure S20: Effect of salt concentrations on CIP-AuNPs formulated with 2 mM CIP; Figure S21: Effect of salt concentrations on CIP-AuNPs formulated with 2.5 mM CIP; Table S1. Spectral assignments for AuNPs, CIP-AuNPs, and free CIP.
