# Supplementary material for: Ciprofloxacin-Loaded Gold Nanoparticles against Antimicrobial Resistance: An In Vivo Assessment
Source: Nanomaterials (Basel). 2021 Nov 22;11(11):3152. doi: 10.3390/nano11113152 (PMC8620493; doi:10.3390/nano11113152)
Supplement: Supplementary file 1 [file nanomaterials-11-03152-s001.zip › nanomaterials-1370409-supplementary.pdf]

Supplementary material

Ciprofloxacin-Loaded Gold Nanoparticles against Antimicrobial Resistance: An In Vivo Assessment

Afrah Nawaz [1], Syed Mohsin Ali [1], Nosheen Fatima Rana [1,*], Tahreem Tanweer [1], Amna Batool [1], Thomas J. Webster [2], Farid Menaa [3], Sundus Riaz [1], Zahra Rehman [1], Farhat Batool [1], Misha Fatima [1], Tuba Maryam [1], Iqra Shafique [1], Abida Saleem [1], Arfa Iqbal [1]

[1] Department of Biomedical Engineering and Sciences, School of Mechanical & Manufacturing Engineering, National University of Science & Technology, Islamabad 44000, Pakistan; anawaz.bmes16smme@student.nust.edu.pk (A.N.); sali.bmes17smme@student.nust.edu.pk (S.M.A.); ttanveer.pg@smme.edu.pk (T.T.); amnabatoolx@gmail.com (A.B.); sundusriaz@parc.gov.pk (S.R.); zrehman.bms20smme@student.nust.edu.pk (Z.R.); farhat.bms20smme@student.nust.edu.pk (F.B.); mfatima.bms20smme@student.nust.edu.pk (M.F.); tuba.bms20smme@student.nust.edu.pk (T.M.); ishafique.phd21smme@student.nust.edu.pk (I.S.); asaleem.phd21smme@student.nust.edu.pk (A.S.); aiqbal.bmes19smme@student.nust.edu.pk (A.I.)

[2] Department of Chemical Engineering, Northeastern University, Boston, MA 02115-5000, USA; websterthomas02@gmail.com

[3] Departments of Internal Medicine and Nanomedicine, California Innovations Corporation, San Diego, CA 92037, USA; dr.fmenaa@gmail.com

* Correspondence: nosheen.fatima@smme.nust.edu.pk; Tel.: +92-519-085-6074; Fax: +92-908-56001

Abstract: Metallic nanoparticles, such as gold nanoparticles (AuNPs), have been extensively studied as drug delivery systems for various therapeutic applications. However, drug-loaded-AuNPs have been rarely explored in-vivo, for their effect on bacteria residing inside tissues. Ciprofloxacin (CIP) is a second generation fluoroquinolone with broad spectrum antibiotic devoid of resistance development. This research is focused on the synthesis and physical characterization of Ciprofloxacin loaded gold nanoparticles (CIP-AuNPs) and their effect on the colonization of *Enterococcus faecalis* in the liver and kidneys of mice. The successfully prepared CIP-AuNPs were stable and exerted enhanced in-vitro antibacterial activity against *E. faecalis* as compared to free CIP. The optimized CIP-AuNPs were administered (500 µg/Kg) once a day via tail vein to infected mice for eight days and were found to be effective in eradicating *E. faecalis* from the host tissues. Moreover, unlike CIP, CIP-AuNPs were non-hemolytic. The CIP-AuNPs showed to be a promising and biocompatible alternative therapeutic for *E. faecalis* induced infections resistant to conventional drugs (e.g., beta-lactams and vancomycin) and should be further investigated.

Keywords: Drug delivery, antibiotics, antimicrobial resistance, gold nanoparticles, Ciprofloxacin, Enterococcus faecalis, liver and kidneys infections, and nanotechnology

1. Experimentation details

1.1. Concentration of AuNPs and CIP-AuNPs

The concentration of AuNPs and CIP-AuNPs was evaluated with the help of Beer's Law.

$A = E \times I \times C$

Where,

A (L mol $^{-1}$ cm $^{-1}$) is the absorbance, E (M^{-1} cm^{-1}) is the molar extinction coefficient, I represent the path length (cm), and C is the concentration (mol).

The UV–visible spectroscopy (UV-vis) absorption spectra were recorded in the range of 300–800 nm. The extinction coefficient was obtained from the standard curve.

1.2. Calculation of unloaded CIP

Beer Lambert's Law was used to calculate unloaded CIP, which says that $A = EcL$

Where A is the absorbance of the CIP-AuNPs solution, E is the extinction coefficient, L is the path length of the cuvette, and c is the concentration of the CIP in the solution.

1.4. Kinetic analysis of the drug release

To unveil the process of drug release from the CIP-AuNPs, numerous mathematical models (zero order, first order, and Higuchi's model) were employed.

To figure out whether the drug dissolution from the CIP-AuNPs was independent of the drug concentration, the zero-order paradigm was applied [1].

$Q_t=Q_0+K_0t$

where Qt is the amount of CIP from CIP-AuNPs dissolved at time t, Q_0 is the initial amount of CIP in the solution (most times, $Q_0 = 0$), and K_0 is the zero-order release constant expressed in units of concentration/time.

The first-order model was applied to find out if the CIP release from the CIP-AuNPs was concentration-dependent [2].

$\text{Log } C= \log C_0 – Kt/2.303$

Where C_0 is the initial concentration of CIP and K is the first-order constant.

The Higuchi model was applied to see if CIP release from CIP-AuNPs was similar to the release mechanism from the matrix and polymeric systems [3].

$Q_t=K_H\, t\ ^1/_2$

Where Q_t is the amount of CIP released at time t and K_H is the release rate constant for the Higuchi model.

The Korsmeyer-Peppas model was also employed to look for the CIP released from the matrix and polymeric systems by using the following equation [3].

$M_t/M_\infty = Kt^n$

Where $Mt/M\infty$ is a fraction of CIP released at time t, K is the CIP release rate constant, and n is the release exponent. For cylindrical-shaped matrices, the n value is used to describe multiple releases. The n value indicates the release mechanism: when $n = 1$, the release rate is independent of time (zero-order, case II transport). $n = 0.5$ stands for Fickian diffusion and when $0.5 < n < 1.0$, diffusion and non-Fickian transport are implicated. Finally, when $n > 1.0$, super case II transport is apparent, and n is the slope value for log ($Mt/M\infty$) versus log time curve.

2. Results

2.1. Elemental chemical composition of AuNPs by EDS

EDX analysis was carried out for elemental mapping and to chemically characterize the NPs, which showed distribution of metallic gold **(Figure S1)**. Due to the specific atomic structure of each element present in the AuNPs and CIP-AuNPs, a specific group of peaks were shown on their spectra for electromagnetic emission [4]. EDAX analysis for CIP-AuNPs showed the presence of Au as the major element in the sample, in addition to carbon, oxygen, chloride and other elements. The respective relevant spectra with strong signals of Au (1.5–2, 9.5–10 and 11-11.5 keV) at characteristic energy were observed. The EDX spectra identified the presence of chloride as a characteristic for CIP-HCl (0- 0.5 keV, 2.5-3 keV). The Na peak (1-1.5 keV) is due to trisodium citrate used as a reducing and capping agent during CIP-AuNPs synthesis. The percentage composition of carbon and oxygen was elevated in the CIP-AuNPs when compared with the EDS spectra of AuNPs from previous studies [5]. A significant overlap from the peaks of Si was observed from 1.5-2 KeV which was due to the silicon substrate on the mounting base.

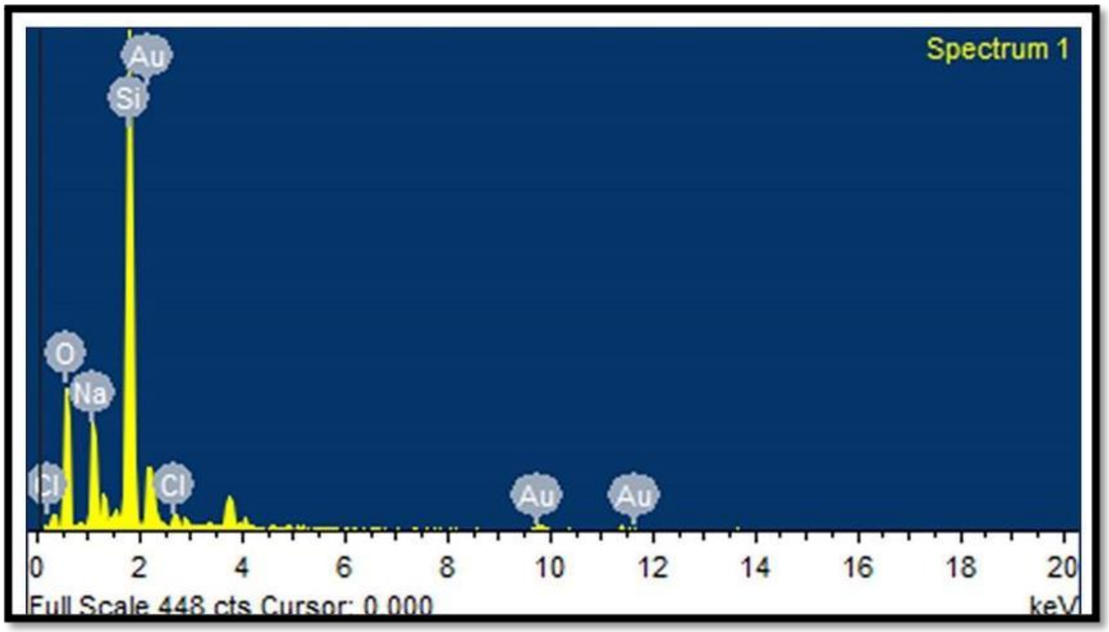

Figure S1. EDX analysis of the CIP-AuNPs

Table S1. Spectral assignments for unloaded AuNPs, CIP-AuNPs and free CIP.

	**Wavenumber (cm^{-1})**	**Bond**	**Functional Group**
**Free CIP**	3410	N-H	Imine
	1655	N-H	Amine
**Unloaded AuNPs**	3320	O-H	Aromatic Alcohol, Phenol compounds
	1634	C=O	Carboxyl
	1381	C-O	
**CIP-AuNPs**	3341	O-H	Aromatic Alcohol, Phenol compounds
	2062	C-H	Carboxyl
	3449	N-H	Imine
	1667	N-H	Amine

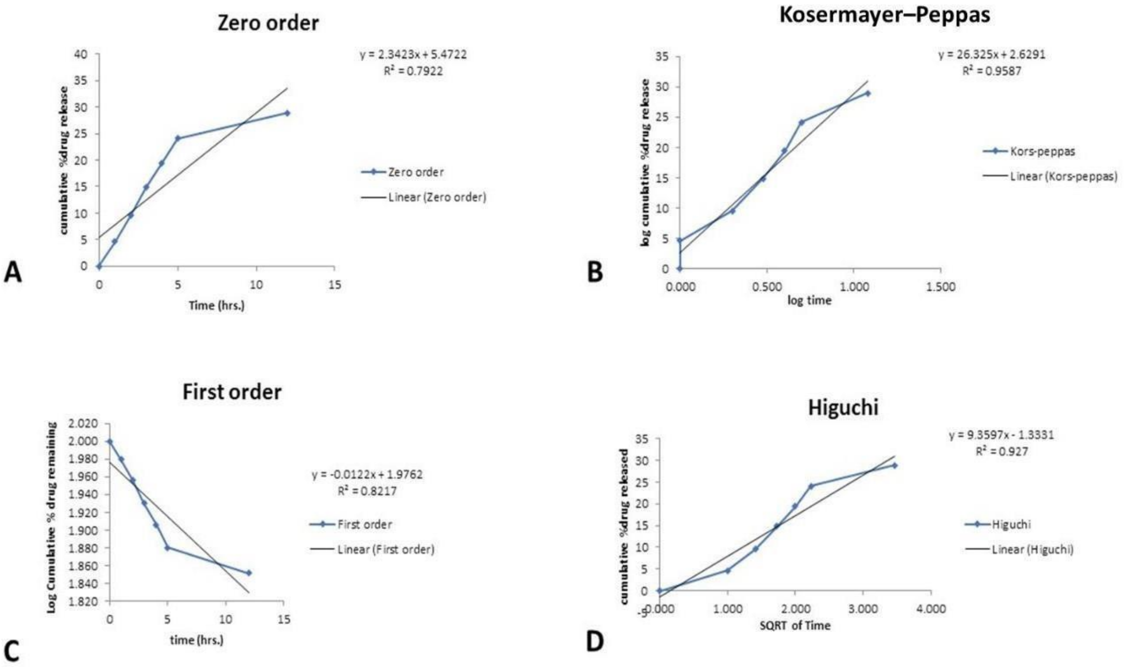

Figure S2: Kinetic analysis of the drug release for CIP-AuNPs (0.5 mM CIP): **(A)** Zero-order kinetics, **(B)** Kosermayer–Peppas plot, **(C)** First-order kinetics, and **(D)** Higuchi kinetics.

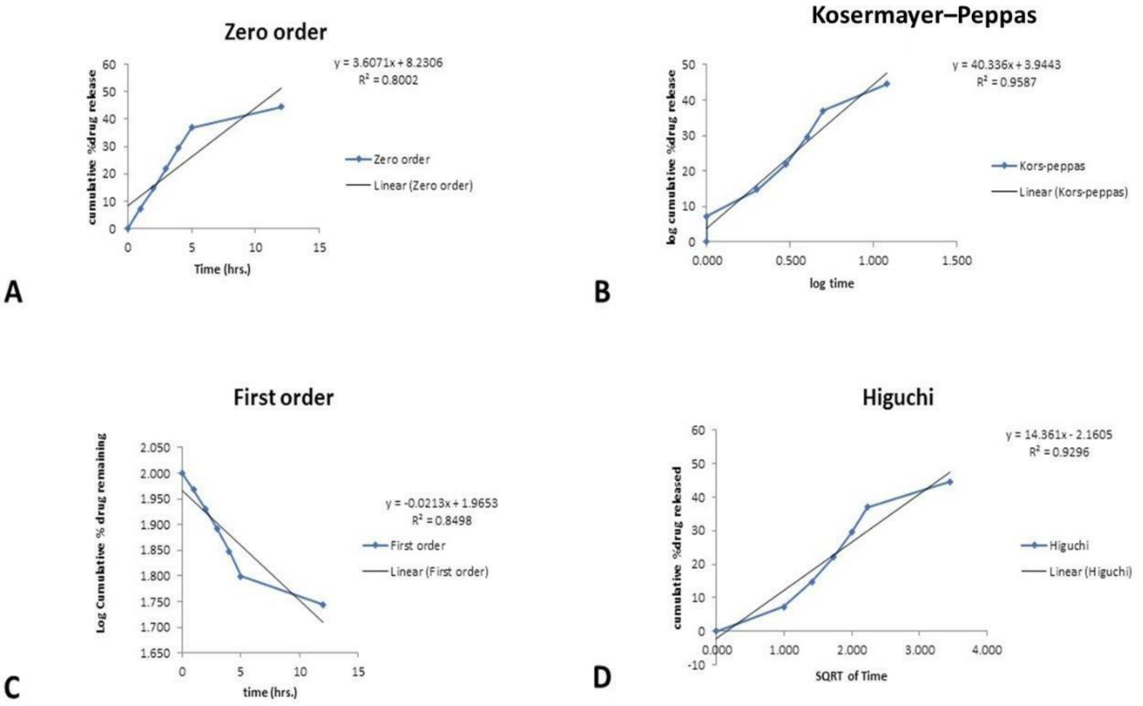

Figure S3: Kinetic analysis of the drug release for CIP-AuNPs (1 mM CIP): (**A**) Zero-order kinetics, (**B**) Kosermayer–Peppas plot, (**C**) First-order kinetics, and (**D**) Higuchi kinetics.

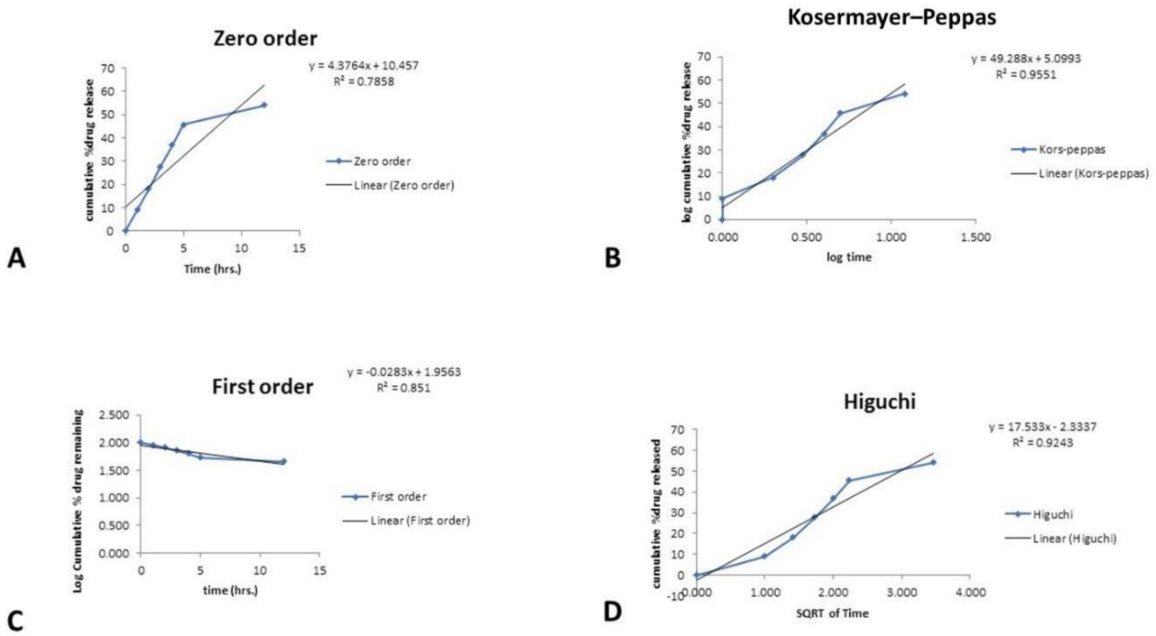

Figure S4: Kinetic analysis of the drug release for CIP-AuNPs (1.5 mM CIP): **(A)** Zero-order kinetics, **(B)** Kosermayer–Peppas plot, **(C)** First-order kinetics, and **(D)** Higuchi kinetics.

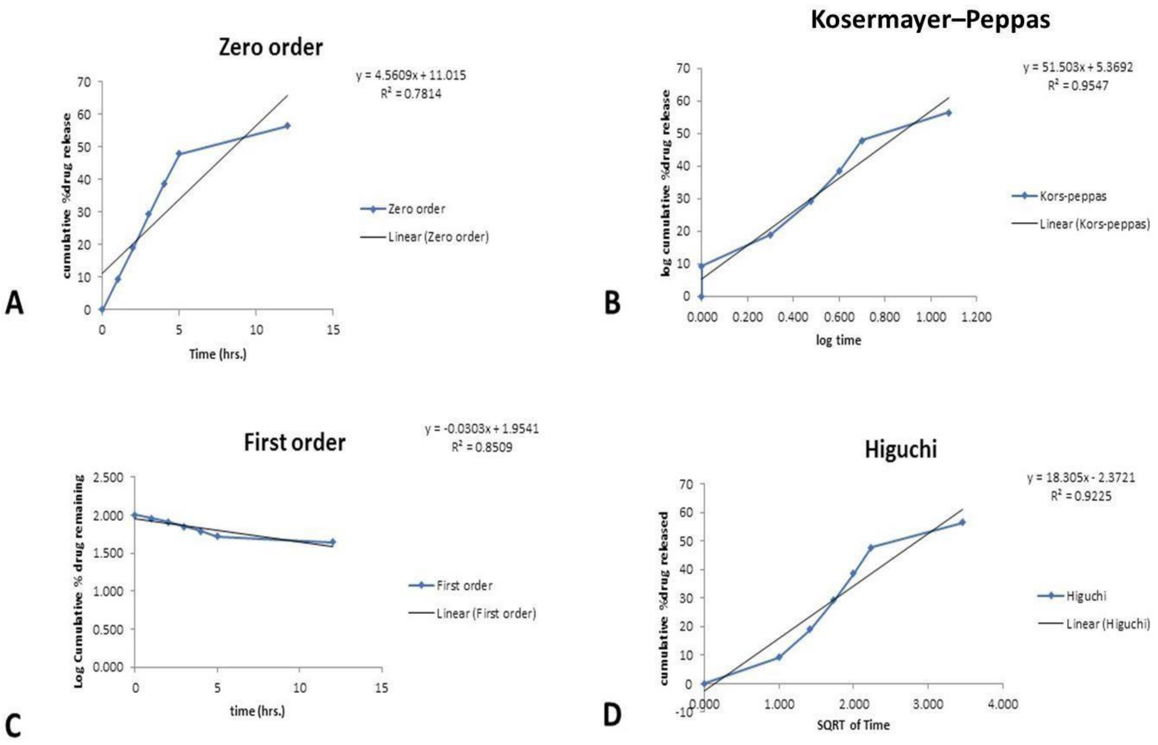

Figure S5: Kinetic analysis of the drug release for CIP-AuNPs (2 mM CIP): **(A)** Zero-order kinetics, **(B)** Kosermayer–Peppas plot, **(C)** First-order kinetics, and **(D)** Higuchi kinetics.

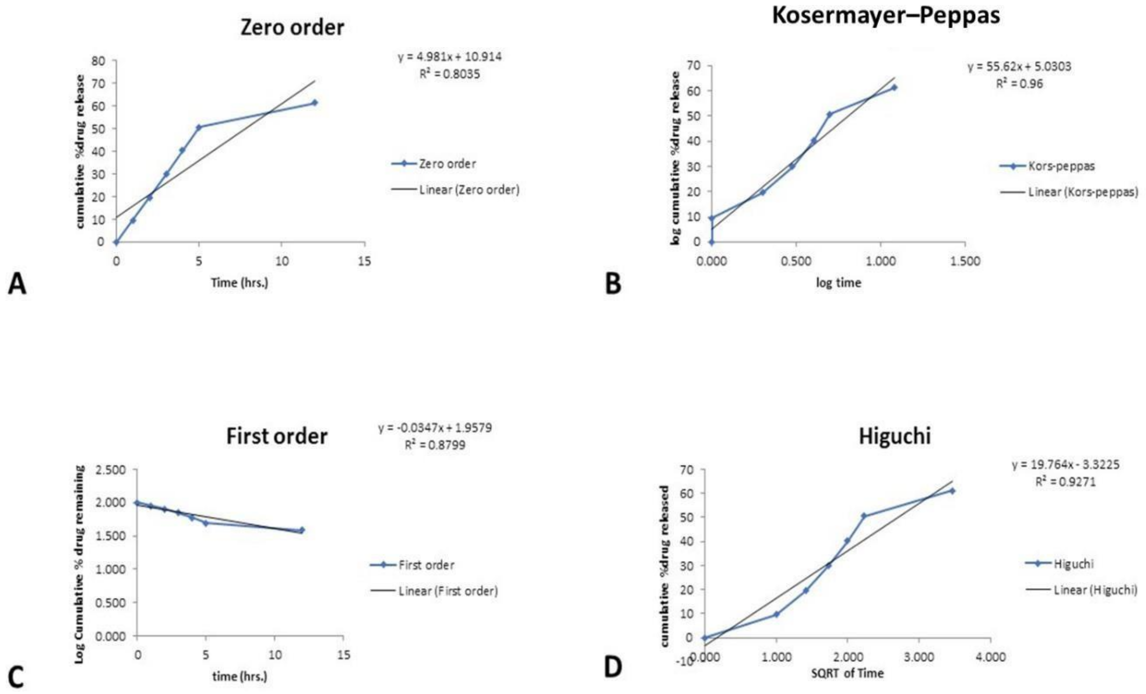

Figure S6: Kinetic analysis of the drug release for CIP-AuNPs (2.5 mM CIP): (**A**) Zero-order kinetics, (**B**) Kosermayer–Peppas plot, (**C**) First-order kinetics, and (**D**) Higuchi kinetics.

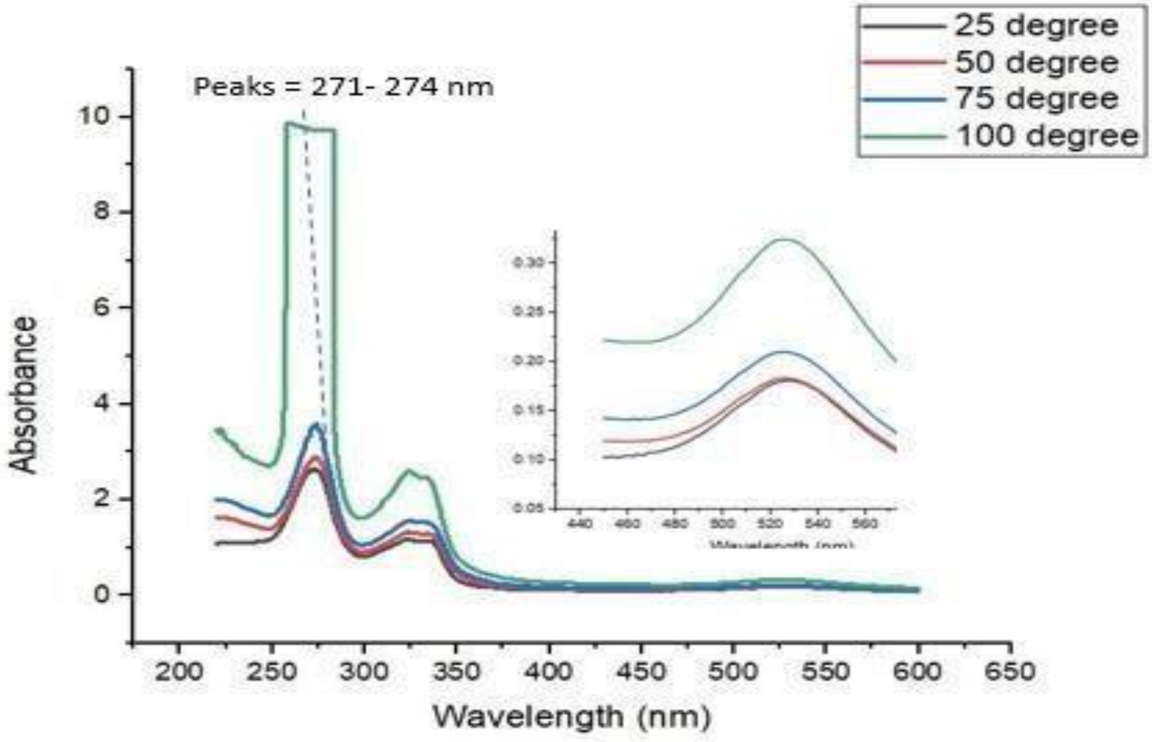

Figure S7: Temperature effect on CIP-AuNPs formulated with 0.5 mM CIP.

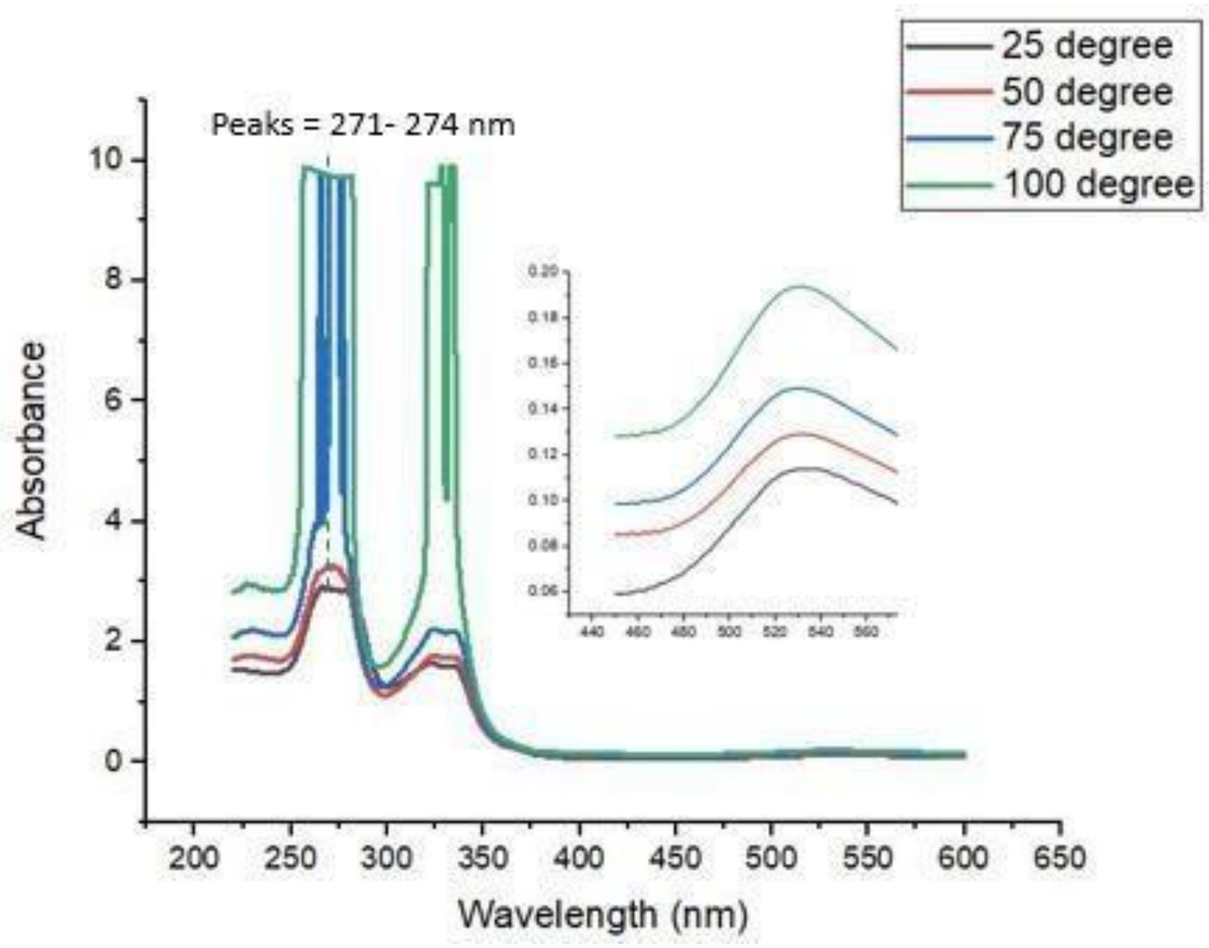

Figure S8: Temperature effect on CIP-AuNPs formulated with 1 mM CIP.

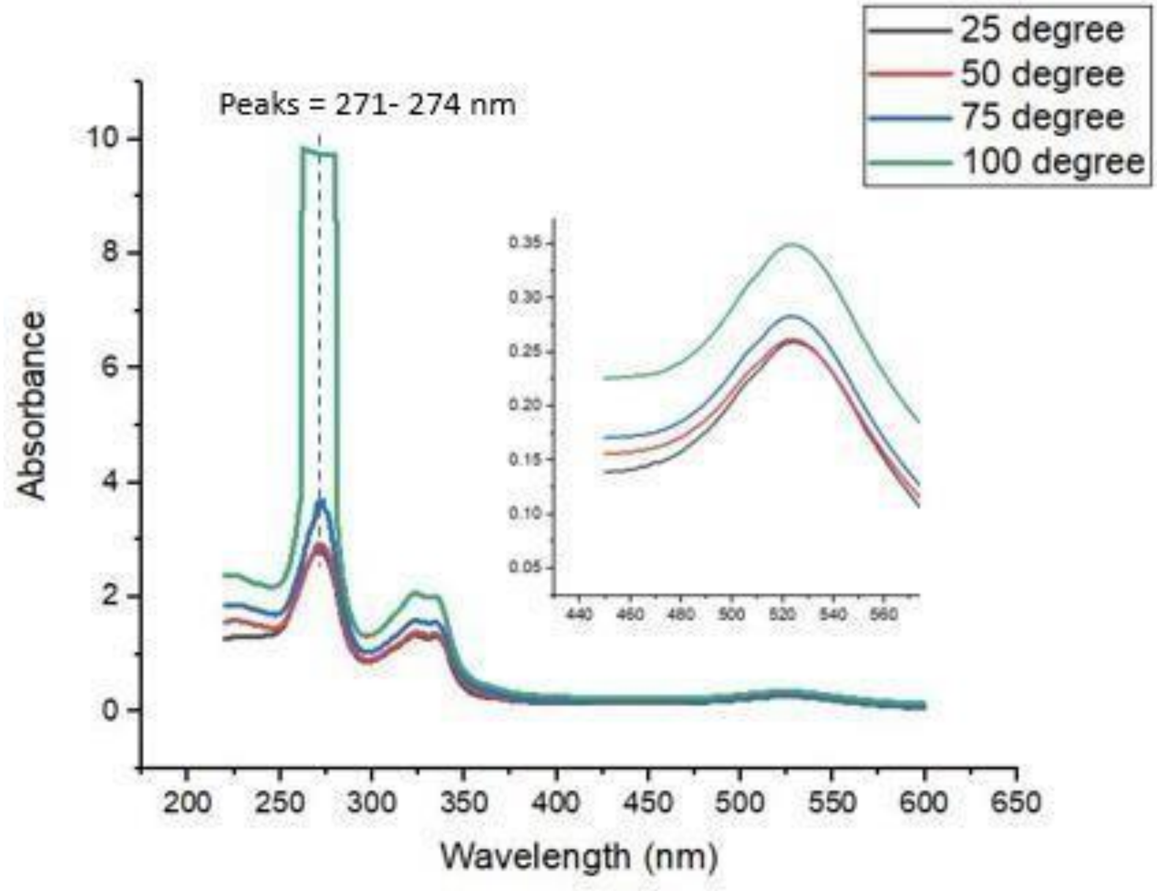

Figure S9: Temperature effect on CIP-AuNPs formulated with 1.5 mM CIP.

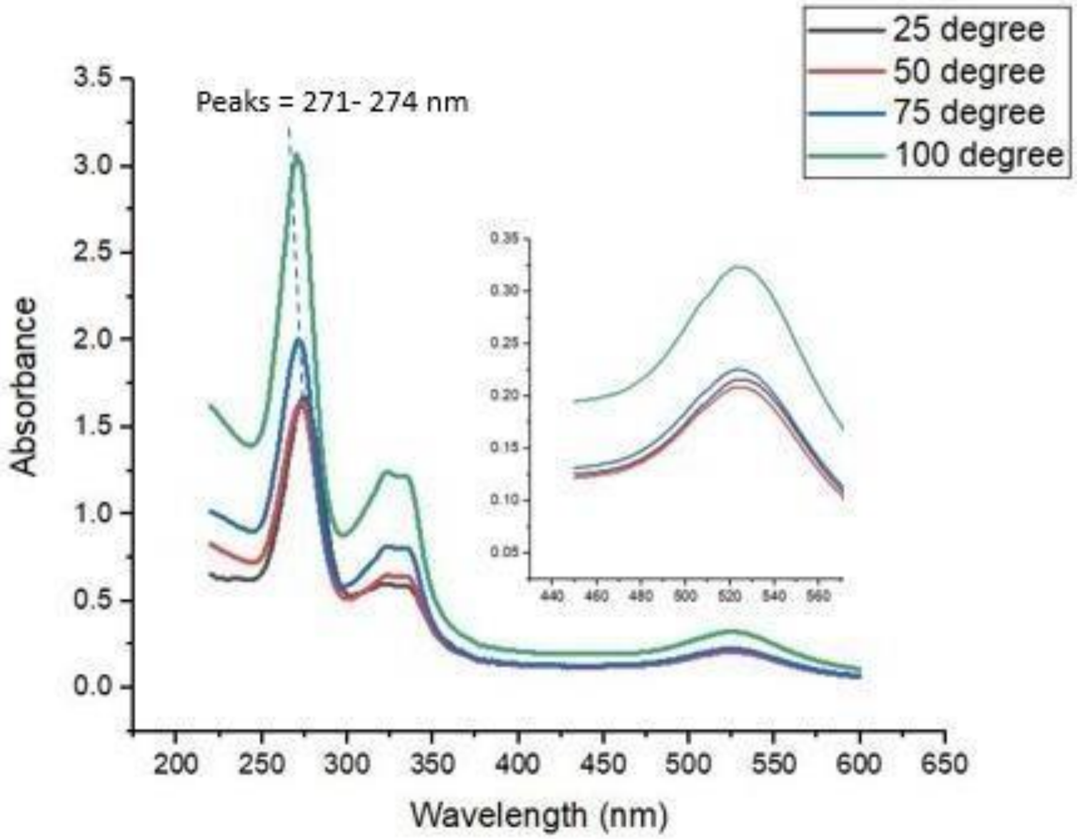

Figure S10. Temperature effect on CIP-AuNPs formulated with a 2 mM CIP concentration.

Temperature Stability (2.5 mM)

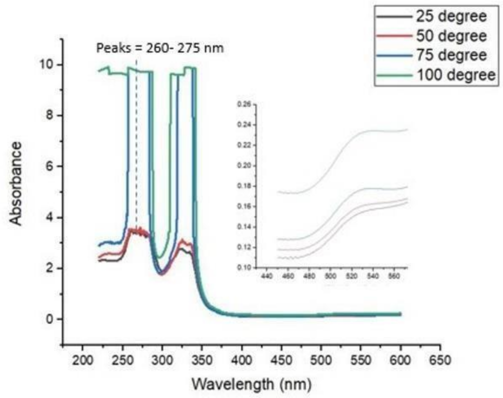

Figure S11: Temperature effect on CIP-AuNPs formulated with 2.5 mM CIP.

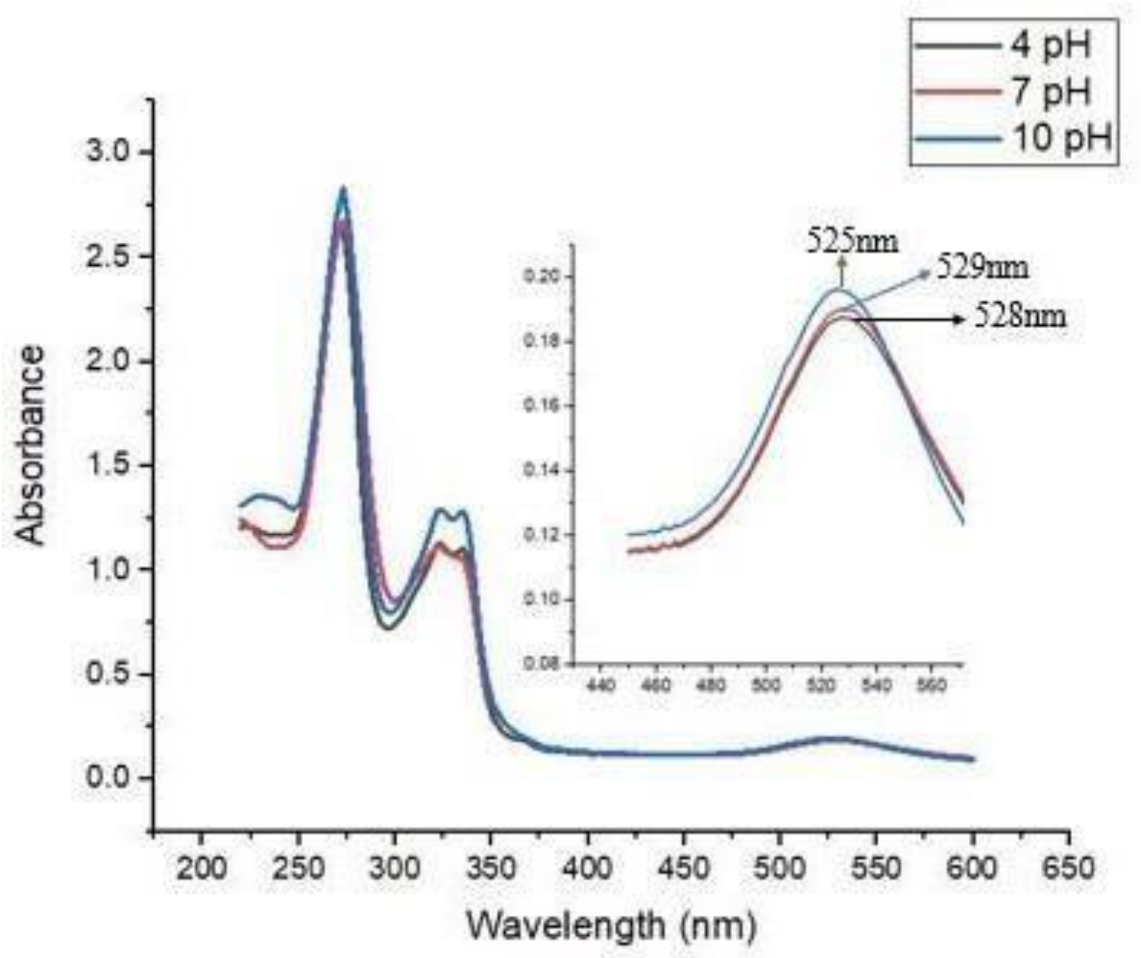

Figure S12: pH effect on CIP-AuNPs formulated with 0.5 mM CIP.

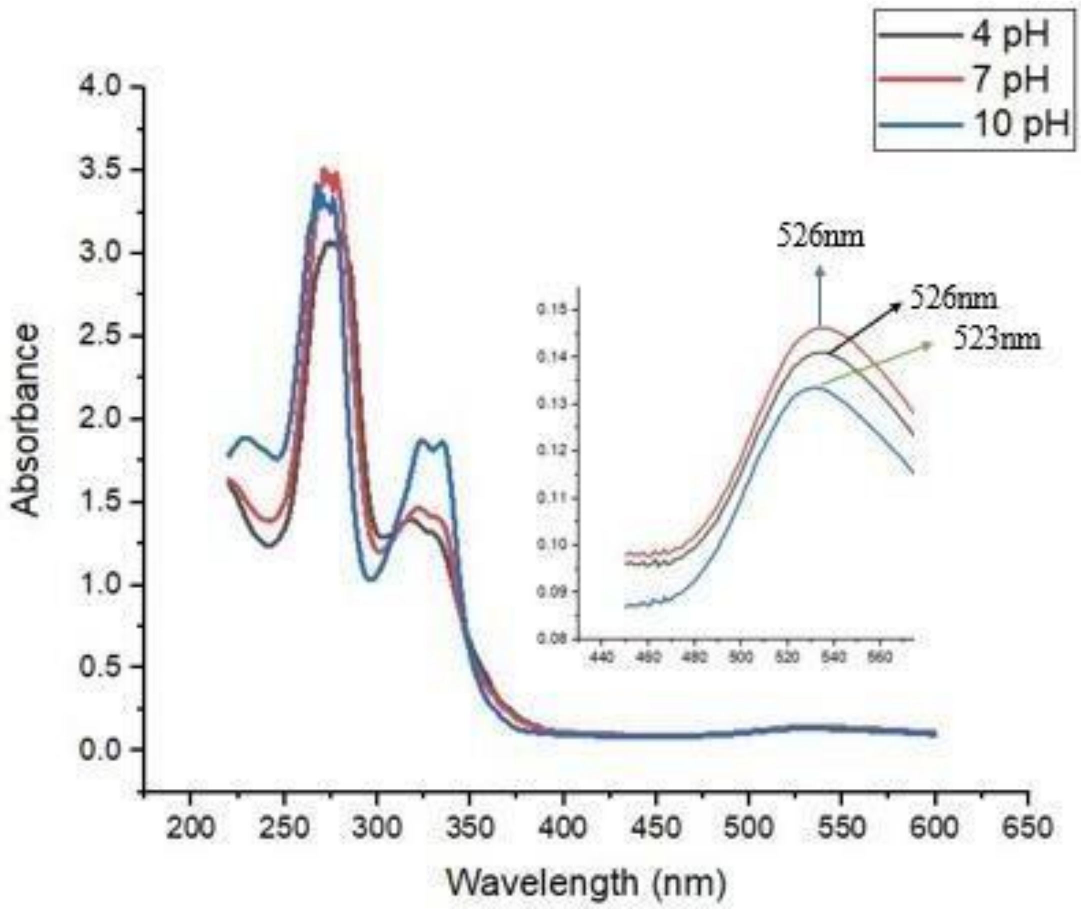

Figure S13: pH effect on CIP-AuNPs formulated with 1 mM CIP.

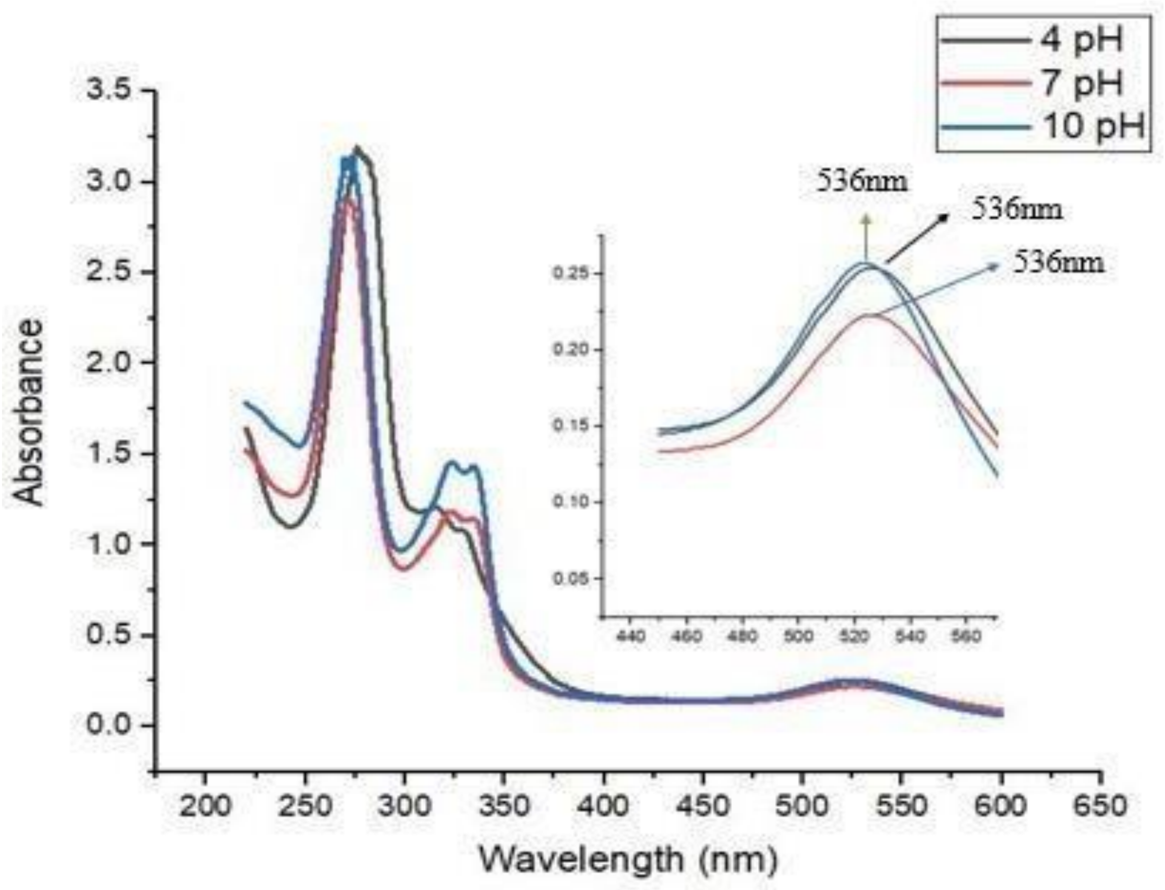

Figure S14: pH effect on CIP-AuNPs formulated with 1.5 mM CIP.

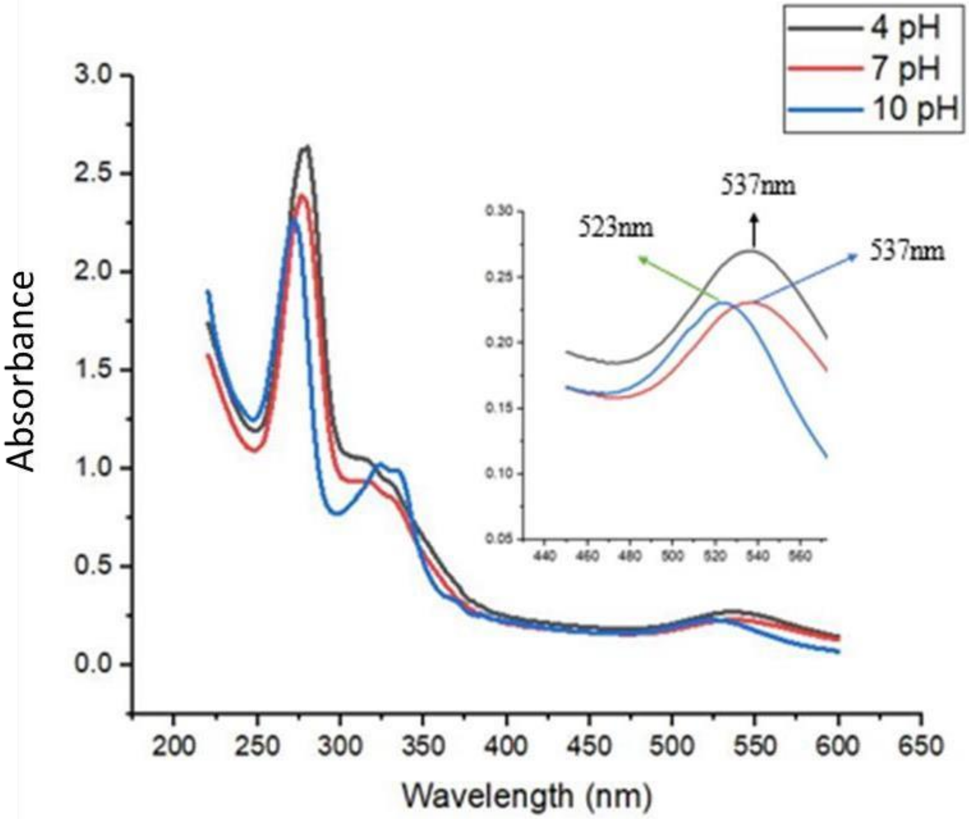

Figure S15. pH effect on CIP-AuNPs formulated with 2mM CIP.

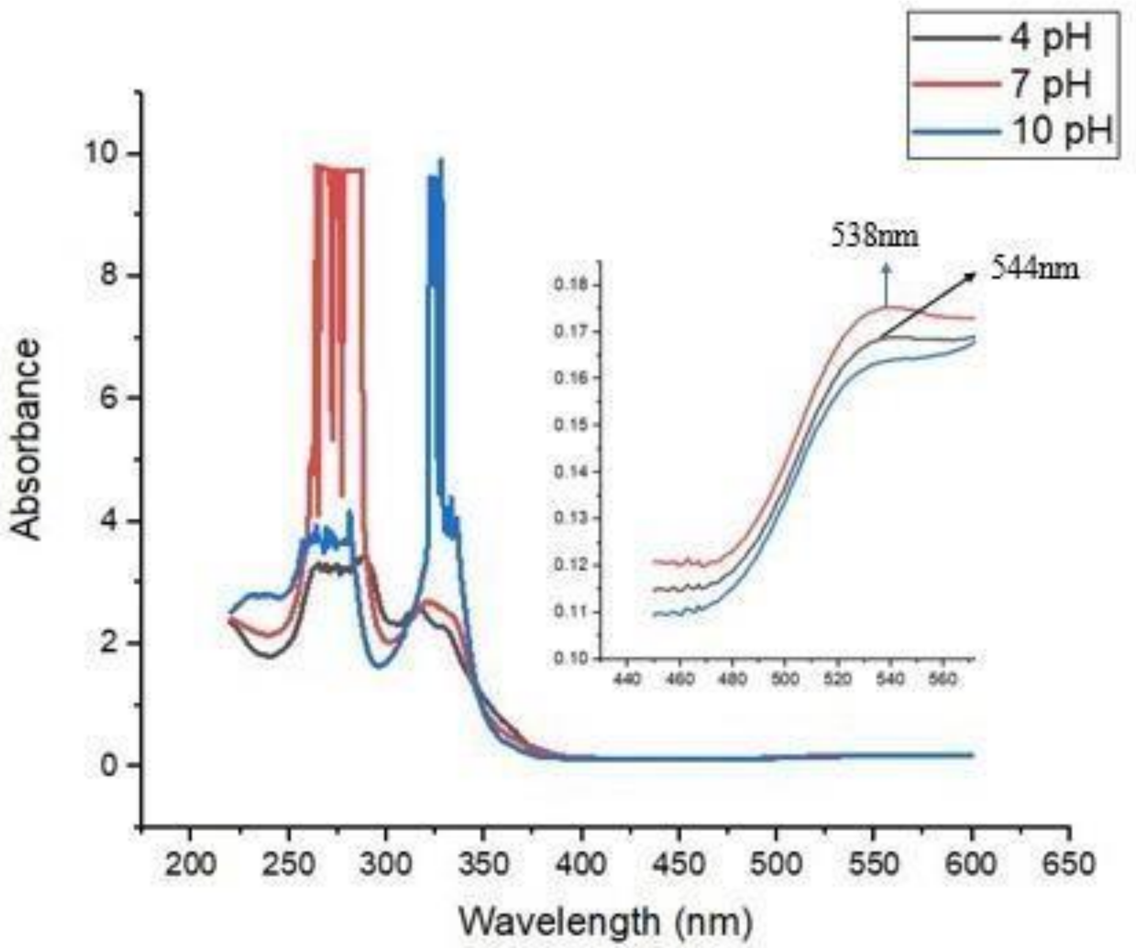

Figure S16: pH effect on CIP-AuNPs formulated with 2.5 mM CIP.

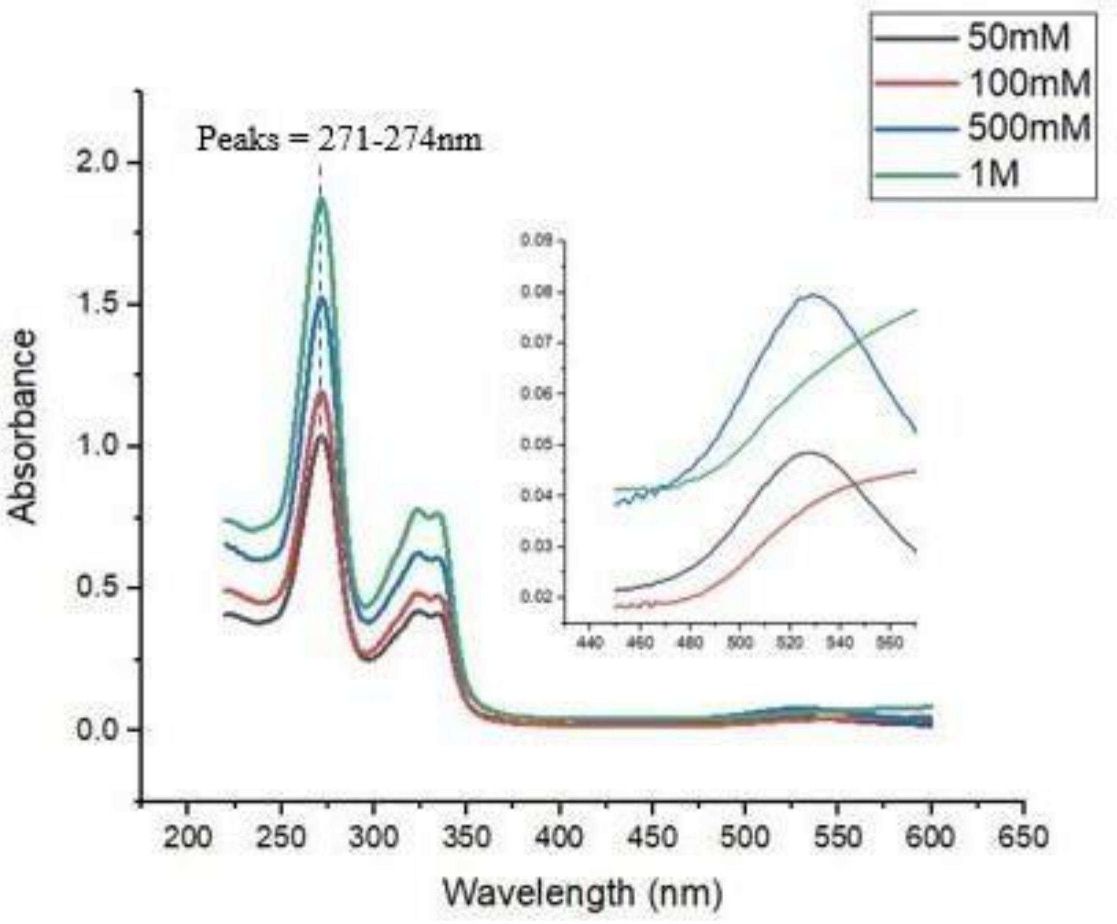

Figure S17: Effect of salt concentrations on CIP-AuNPs formulated with 0.5 mM CIP.

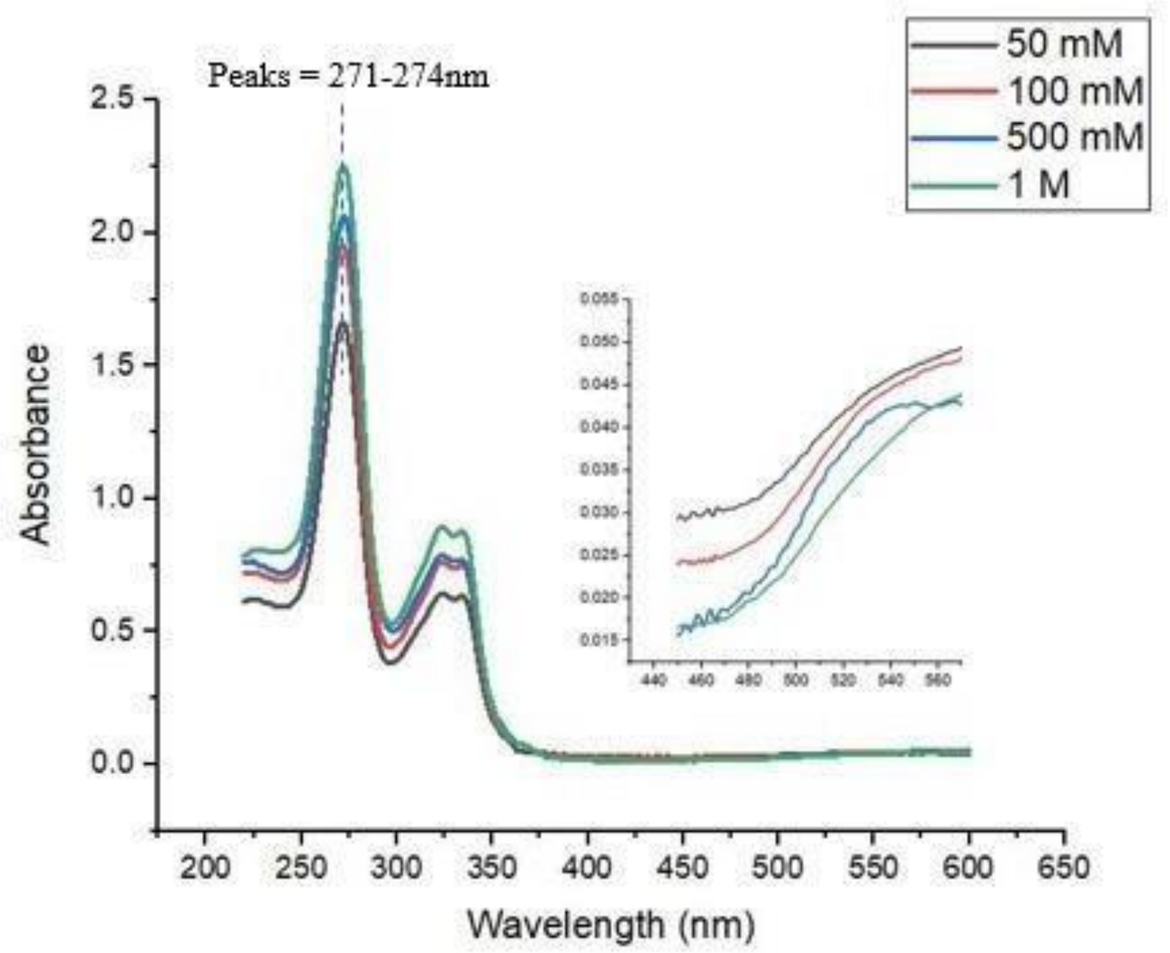

Figure S18: Effect of salt concentrations on CIP-AuNPs formulated with 1 mM CIP.

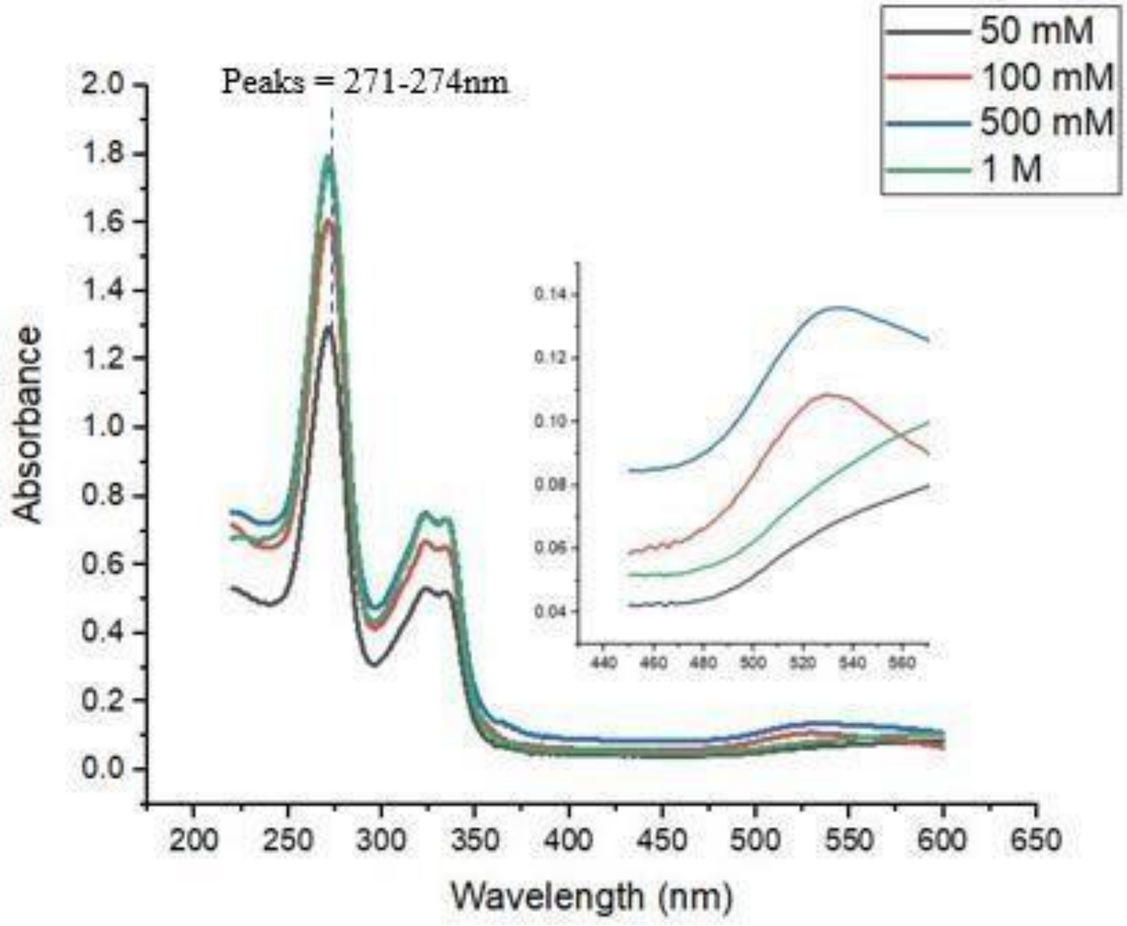

Figure S19: Effect of salt concentrations on CIP-AuNPs formulated with 1.5 mM CIP.

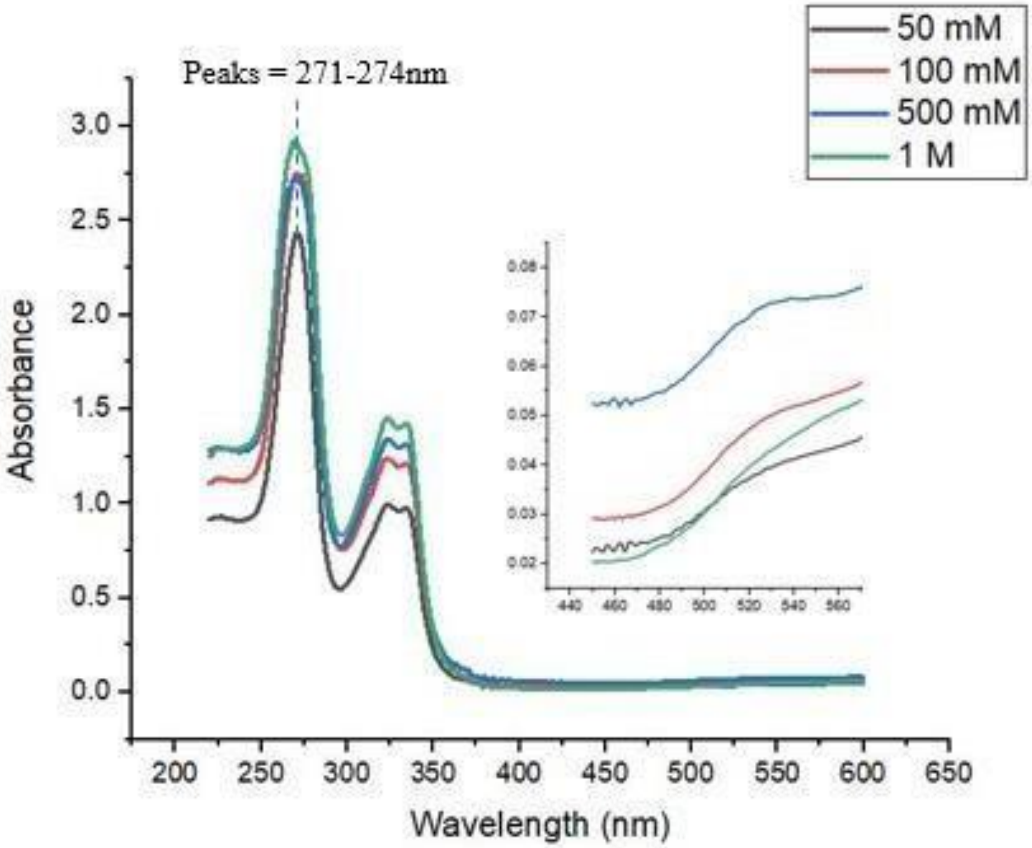

Figure S20. Salt (NaCl) concentrations effect on CIP-AuNPs formulated with 2 mM CIP.

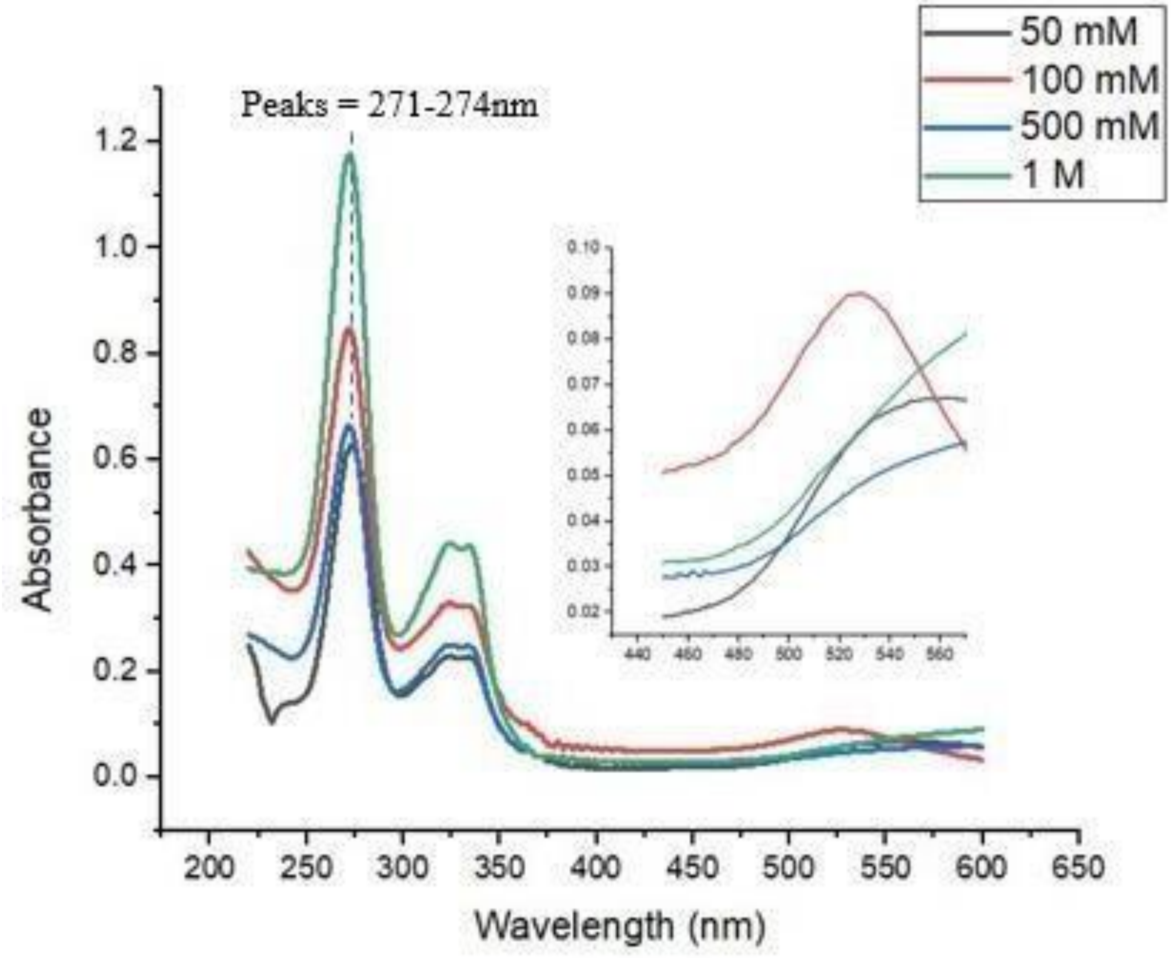

Figure S21: Effect of salt concentrations on CIP-AuNPs formulated with 2.5 mM CIP.

3. References

1 Dash, S., Murthy, P. N., Nath, L., & Chowdhury, P. (2010). Kinetic modeling on drug release from controlled drug delivery systems. Acta Pol Pharm, 67(3), 217-223.

2 Higuchi, T. (1963). Mechanism of sustained-action medication. Theoretical analysis of rate of release of solid drugs dispersed in solid matrices. *Journal of pharmaceutical sciences*, *52*(12), 1145-1149.

3 Bettini, R., Catellani, P. L., Santi, P., Massimo, G., Peppas, N. A., & Colombo, P. (2001). Translocation of drug particles in HPMC matrix gel layer: effect of drug solubility and influence on release rate. *Journal of Controlled Release*, *70*(3), 383-391.

4 Bell, D. C., & Garratt-Reed, A. J. (2003). *Energy dispersive X-ray analysis in the electron microscope* (Vol.49). Garland Science.

5 Qian, L., Su, W., Wang, Y., Dang, M., Zhang, W., & Wang, C. (2019). Synthesis and characterization of gold nanoparticles from aqueous leaf extract of Alternanthera sessilis and its anticancer activity on cervical cancer cells (HeLa). *Artificial cells, nanomedicine, and biotechnology*, *47*(1), 1173-1180.
